# Dim light in the evening causes coordinated realignment of circadian rhythms, sleep, and short-term memory

**DOI:** 10.1073/pnas.2101591118

**Published:** 2021-09-23

**Authors:** Shu K. E. Tam, Laurence A. Brown, Tatiana S. Wilson, Selma Tir, Angus S. Fisk, Carina A. Pothecary, Vincent van der Vinne, Russell G. Foster, Vladyslav V. Vyazovskiy, David M. Bannerman, Mary E. Harrington, Stuart N. Peirson

**Affiliations:** ^a^Sleep and Circadian Neuroscience Institute, Nuffield Department of Clinical Neurosciences, University of Oxford, Oxford OX1 3QU, United Kingdom;; ^b^Research Support Team, IT Services, University of Oxford, Oxford OX1 2JD, United Kingdom;; ^c^Neuroscience Program, Smith College, Northampton, MA 01063;; ^d^Department of Physiology, Anatomy and Genetics, University of Oxford, Oxford OX1 3PT, United Kingdom;; ^e^Behavioural Neuroscience Unit, Department of Experimental Psychology, University of Oxford, Oxford OX1 3TA, United Kingdom

**Keywords:** dim light in the evening, long-day photoperiod, melanopsin, circadian rhythms, short-term memory

## Abstract

Light provides the primary signal for entraining circadian rhythms to the day/night cycle. In addition to rods and cones, the retina contains a small population of photosensitive retinal ganglion cells (pRGCs) expressing the photopigment melanopsin (OPN4). Concerns have been raised that exposure to dim artificial lighting in the evening (DLE) may perturb circadian rhythms and sleep patterns, and OPN4 is presumed to mediate these effects. Here, we examine the effects of 4-h, 20-lux DLE on circadian physiology and behavior in mice and the role of OPN4 in these responses. We show that 2 wk of DLE induces a phase delay of ∼2 to 3 h in mice, comparable to that reported in humans. DLE-induced phase shifts are unaffected in *Opn4*^−/−^ mice, indicating that rods and cones are capable of driving these responses in the absence of melanopsin. DLE delays molecular clock rhythms in the heart, liver, adrenal gland, and dorsal hippocampus. It also reverses short-term recognition memory performance, which is associated with changes in preceding sleep history. In addition, DLE modifies patterns of hypothalamic and cortical cFos signals, a molecular correlate of recent neuronal activity. Together, our data show that DLE causes coordinated realignment of circadian rhythms, sleep patterns, and short-term memory process in mice. These effects are particularly relevant as DLE conditions―due to artificial light exposure―are experienced by the majority of the populace on a daily basis.

## Significance

In modern societies, people are regularly exposed to artificial light (e.g., light-emitting electronic devices). Dim light in the evening (DLE) imposes an artificial extension of the solar day, increasing our alertness before bedtime, delaying melatonin timing and sleep onset, and increasing sleepiness in the next morning. Using laboratory mice as a model organism, we show that 2 wk of 4-h, 20-lux DLE postpones rest–activity rhythms, delays molecular rhythms in the brain and body, and reverses the diurnal pattern of short-term memory performance. These results highlight the biological impact of DLE and emphasize the need to optimize our evening light exposure if we are to avoid shifting our biological clocks.

Light exerts profound effects on physiology and behavior, synchronizing biological rhythms to the light/dark cycle (LD) as well as directly modulating alertness and sleep (1, 2). In mammals, light detected by the eye is the primary time cue synchronizing circadian rhythms of activity and rest, a process termed entrainment. Exposure to light at dawn and dusk plays a key role, adjusting the phase of the master circadian clock in the hypothalamic suprachiasmatic nuclei (SCN) (3–5). Studies on the photoreceptors mediating circadian entrainment led to the identification of a distinct photoreceptor system consisting of a subset of photosensitive retinal ganglion cells (pRGCs) expressing the photopigment melanopsin (OPN4) (6, 7). These cells have a peak sensitivity at ∼480 nm (8, 9), hence differing from the classical visual system, which in humans is most sensitive to light at ∼555 nm, corresponding to the red and green cones of the fovea (10). In addition to modulating image-forming responses via local retinal circuitry, OPN4-expressing pRGC axons project to the SCN and different brain areas, setting the circadian clock and driving nonvisual responses to light (5, 7, 11).

How does the mammalian brain adapt to changes in daylength? In humans, exposure to long-day photoperiods delays melatonin onset but advances melatonin offset, hence compressing the internal biological night, relative to short-day photoperiods; this is observed in laboratory studies (12, 13) as well as under naturalistic conditions (14, 15). In laboratory mice, the onset and offset of wheel-running activity change dynamically in response to daylength (16). Long-day photoperiods also cause functional reorganization in the SCN. In vivo multiunit recording in mice shows that 16-h light/8-h dark cycles (16:8 LD) weakens phase clustering of SCN neurons (17). Similarly, PERIOD2::LUCIFERASE bioluminescence signals in the mouse dorsal versus ventral SCN are dissociated after 20:4 LD (18). Weakened intercellular coupling in the SCN reflects a form of plasticity, enhancing adaptability of the circadian system to an increase in daylength (19). In addition, 19:5 LD reduces the number of dopamine neurons in the hypothalamus, increasing behavioral immobility and decreasing exploratory activity in rats (20) and mice (21); seasonal variation in photoperiod is also associated with changes in dopamine levels in the human midbrain (22). In the mouse hippocampus, molecular rhythms such as *Per1*,*2* and *Cry1*,*2* are blunted under 20:4 LD (23); however, the consequence is complex: it improves object and spatial discrimination in the spontaneous recognition memory task but disrupts context discrimination in the fear conditioning task (23).

Aberrant lighting at night may lead to disrupted circadian rhythms and sleep, which are associated with many adverse health outcomes, including impaired concentration and performance, mood disturbances, metabolic diseases, cardiovascular and neurological disorders, and cancer (24–26). Numerous studies have characterized the disruptive effects of dim light at night (DLAN) on metabolic and mood-related processes in rodents. In these studies, animals were exposed to dim light for the entire night (27–35). As such, DLAN is highly relevant to conditions in which low-level light exposure continues throughout the night, such as light pollution. However, DLAN is somewhat different from exposure to artificial electrical lighting as experienced by the majority of the populace, who typically experience higher light levels during the day (though lower than natural daylight) but dim light for a short period in the evening (DLE) (14, 15, 36, 37). In humans, DLE exposure delays melatonin rhythms and sleep timing (14, 15, 37) and reduces alertness on the subsequent day (36); these phase-delaying effects of DLE on the circadian system are found under both natural summer (14) and winter photoperiods (15). As such, DLE combines features of both long-day photoperiods and DLAN. While similar to a long-day photoperiod, the extended light phase is of a lower light intensity and may exert different effects in comparison with the higher light levels during the day. Conversely, unlike DLAN, under DLE the evening light exposure only occurs at the start of the biological night when the circadian system is most sensitive to light-induced phase delays (38).

Although the effects of long-day photoperiods (12–23) and DLAN (27–35) on circadian physiology and behavior have been extensively studied, the effects of DLE—as produced by artificial light exposure—have received less attention. Here, we investigate the effects of 2 wk of DLE in laboratory mice and the role of OPN4 in mediating these responses. Our choice of dim-light level and duration was based upon human studies conducted in nonlaboratory settings (14, 15, 36, 37), which reported that ∼3 to 4 h of ∼20 to 30 lx artificial lighting exposure increased alertness before bedtime, delayed melatonin timing and sleep onset, and increased sleepiness in the morning. Despite their nocturnality, the mouse phase response curve (PRC) is broadly similar to the human PRC: in both species, light presented during the early night delays circadian rhythms, whereas light presented later at night or early in the morning causes phase advances (5, 38, 39). Our DLE protocol comprises a 12-h light phase at 200 lx, a 4-h evening light period at 20 lx, and an 8-h dark phase. Here, we characterize the effects of 4-h, 20-lux DLE on a) locomotor activity rhythms, b) sleep patterns, c) molecular clocks in peripheral tissues, d) short-term memory process, and e) brain cFos signals.

## Results

### Locomotor Activity Rhythms Are Phase Delayed under DLE.

**Fig. 1.**
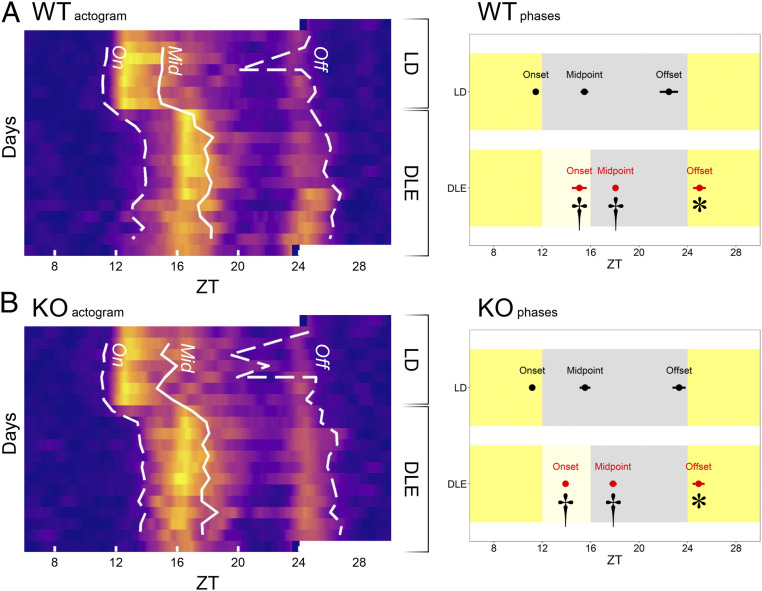
Phase-delaying effects of DLE on PIR locomotor activity (*n* = 12 WT and 12 *Opn4*^−/−^ mice). Data from WT are shown in *A* and data from *Opn4*^−/−^ mice in panel *B*. Actograms on *Left* show the group-averaged locomotor activity traces under LD (days 1 to 7) and DLE (days 8 to 20). ZTs on *x*-axes are plotted from ZT6 to ZT30. Brighter colors indicate higher activity levels. White dashed lines (On and Off) and solid lines (Mid) indicate activity onsets, offsets, and midpoints of the group-averaged actograms. Temporal resolution of the recording was 10 s. Plots on *Right* show means (± SEM) of activity onsets, midpoints, and offsets. ZTs on *x*-axes are plotted from ZT6 to ZT30 (all SE bars are plotted but some imperceptible on the 24-h scale). All three phase markers were delayed under DLE in both WT and *Opn4*^−/−^ mice (main effects of Lighting: *Ps < 0.05; ^†^*P*s < 0.001).

To examine the effects of 20-lux DLE during Zeitgeber times (ZT)12 to 16 on circadian rhythms, locomotor activity phases (onset, midpoint, and offset) were determined from passive infrared sensor (PIR) recording across each 24-h cycle. Among the three phase markers, activity offset is known to show the highest variability (40); this is in agreement with our data under LD. Under DLE, C57BL/6 wild-type (WT) mice exhibited changes in alignment of all three parameters with respect to the light phase (Fig. 1*A*). More specifically, activity onsets were delayed by 3.59 ± 0.46 h [main effects of Lighting *F*(1, 10) = 64.31, *P* < 0.001], whereas activity midpoints and offsets were delayed by 2.57 ± 0.37 and 2.52 ± 0.83 h [*F*(1, 10) = 43.94, *P* < 0.001 and *F*(1, 10) = 10.64, *P* = 0.009, respectively]. Changes in activity midpoints and offsets, which occurred hours after the DLE, suggest that these are not the acute effect of activity suppression by DLE. Furthermore, under constant darkness (DD), activity rhythms in mice with prior exposure to DLE free ran at later clock times than mice with prior exposure to LD (*n* = 8 *Albumin*-*Cre*;*Dbp*^KI/+^ mice per group as described in *Molecular Rhythms Are Delayed under DLE*), with an overall phase difference of ∼4 h [main effects of Prior Lighting: *F*(1, 14) = 6.77, *P* = 0.021; *F*(1, 14) = 6.30, *P* = 0.025; and *F*(1, 14) = 6.20, *P* = 0.026, for onsets, midpoints, and offsets, respectively]. The persistence of DLE-induced phase delay under DD confirmed that these effects were not the result of the suppression of locomotor activity by light.

As the oestrous cycle in female mice (which lasts for 4 to 5 d) has been shown to affect wheel-running activity rhythms (41), we assessed the possibility that the effects of DLE on PIR activity rhythms could vary between sexes. However, there was no Lighting × Sex interaction, indicating that DLE exerted comparable effects in both sexes [Lighting × Sex interactions *F*(1, 10) = 1.66, *P* = 0.23; *F*(1, 10) = 0.06, *P* = 0.81; and *F*(1, 10) = 2.68, *P* = 0.13, for onsets, midpoints, and offsets, respectively].

### DLE-Induced Phase Delays Are Unaffected by OPN4 Deficiency.

Similar DLE-induced phase delays were observed in OPN4-deficient (*Opn4*^−/−^) mice of both sexes. Under DLE, activity onsets, midpoints, and offsets of *Opn4*^−/−^ mice were delayed by 2.74 ± 0.18, 2.32 ± 0.95, and 1.61 ± 0.61 h [main effects of Lighting *F*(1, 10) = 215.25, *P* < 0.001; *F*(1, 10) = 87.14, *P* < 0.001; and *F*(1, 10) = 7.53, *P* = 0.021, respectively; Fig. 1*B*]. Although the size of phase shifts appeared smaller in *Opn4*^−/−^ mice, comparisons between WT controls and *Opn4*^−/−^ mice indicated that DLE effects were statistically indistinguishable between genotypes (main effects of Genotype *P*s > 0.08; Lighting × Genotype interactions *P*s > 0.09). Thus, like other nonvisual responses to light (42–44), the conventional retinal circuitry can support DLE-induced phase delays in the absence of OPN4. Under DLE, the power of ∼24 h rhythms persisted in both WT and *Opn4*^−/−^ mice; however, there was an increase in oscillatory power at ∼8 h in both groups as revealed by continuous wavelet transform (45) (*SI Appendix*, Fig. S1). This is likely to be an artifact of the shortened, 8-h night under DLE rather than reflecting an increase in ultradian rhythms per se.

### Sleep Patterns Are Realigned under DLE.

**Fig. 2.**
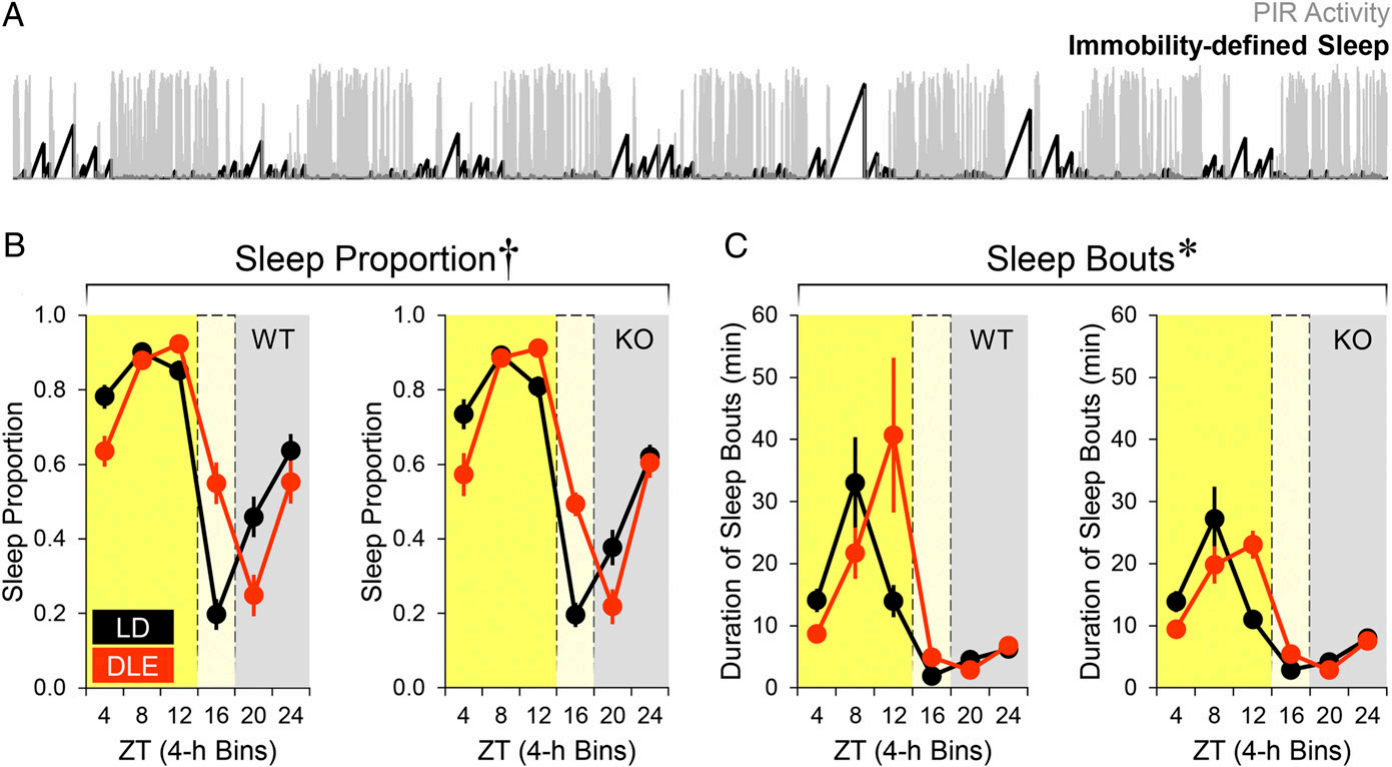
Effects of DLE on immobility-defined sleep (*n* = 12 WT and 12 *Opn4*^−/−^ mice). Sleep was defined as sustained immobility (i.e., PIR activity = 0) for four consecutive 10-s bins or longer (*A*; the black trace shows 7 d of PIR-immobility-defined sleep under LD from one WT animal). (*B* and *C*) Sleep proportion and duration of sleep bouts in 4-h bins (Lighting × Time of Day interactions: *P < 0.025; ^†^*P* < 0.001).

To examine changes in sleep patterns, for each 10-s bin of the PIR recording, the mouse’s behavioral state was assigned as either awake (0) or asleep (1), where “1” was defined as sustained PIR inactivity for at least four uninterrupted 10-s bins; if this was not the case, a value of “0” was assigned (Fig. 2*A*). Sleep proportion was calculated as the number of bins assigned with a value of “1” divided by the total number of 10-s bins during that time window; the duration of a sleep bout was defined as the number of uninterrupted 10-s bins assigned with a value of “1.” It has been validated that ≥40 s immobility is highly correlated with electroencephalogram (EEG)-defined sleep times across the 24-h day, with Pearson’s *r* ≥ 0.94 (46–48).

Total immobility-defined sleep times were unaffected under DLE (*SI Appendix*, Table S1). However, there were changes in the alignment of sleep patterns under DLE, both in terms of sleep proportion [Lighting × Time of Day interaction in WT: Greenhouse–Geisser corrected *F*_*Ɛ*_(2,29) = 26.03, *P* < 0.001; Lighting × Time of Day interaction in *Opn4*^−/−^: *F*_*Ɛ*_(3,33) = 24.17, *P* < 0.001; Fig. 2*B*] as well as duration of sleep bouts [Lighting × Time of Day interaction in WT: *F*_*Ɛ*_(1,14) = 6.40; *P* = 0.017; Lighting × Time of Day interaction in *Opn4*^−/−^: *F*_*Ɛ*_(1,19) = 14.10, *P* < 0.001; Fig. 2*C*]. Crucially, under DLE, sleep proportion was reduced and sleep bouts were shortened in the first 4 h of the light phase (simple effects of Lighting from ZT0 to 4 *P*s < 0.005). Thus, DLE promoted sleep from ZT12 to 16 and postponed the accumulation of sleep pressure in the next morning. Comparisons between WT and *Opn4*^−/−^ mice did not reveal any genotype difference (main effects of Genotype *P*s > 0.08; Lighting × Genotype interactions *P*s > 0.1; Lighting × Time of Day × Genotype interactions *P*s > 0.2).

As sleep and inactivity are often accompanied by body cooling (49), we examined if there was any change in skin temperature (*T*_skin_) using infrared thermography (*SI Appendix*, Fig. S2*A*) as described in our previous study (50). *T*_skin_ provides an estimate of changes in core body temperature over time (50). The phase of *T*_skin_ offset was delayed under DLE (*SI Appendix*, Fig. S2*B*), corresponding to the delayed PIR activity rhythm and sleep profile. In addition, there was a 2% drop in *T*_skin_ (∼0.65 °C) during the 4-h DLE period as well as a 0.5 to 1% reduction in *T*_skin_ (∼0.15 to 0.35 °C) in the light phase (*SI Appendix*, Fig. S2*C*).

### DLE Effects Are Weaker Than Effects of a Long Day.

When the intensity of the 4-h evening light is the same as the prevailing light phase, this would become a long-day photoperiod. We compared locomotor activity rhythms and sleep under 16-h day/8-h night cycles (16:8 LD) versus DLE. Under 16:8 LD, activity onsets and midpoints were further delayed by 2.21 ± 0.49 and 0.99 ± 0.18 h relative to DLE [main effects of Lighting *F*(1, 22) = 20.80, *P* < 0.001 and *F*(1, 22) = 29.81, *P* < 0.001, respectively]. The difference in activity offsets under 16:8 LD versus DLE, 0.64 ± 0.36 h, was not significant (*P* > 0.09). Comparisons between WT and *Opn4*^−/−^ mice did not reveal any genotype difference in phasing (main effects of Genotype *P*s > 0.4; Lighting × Genotype interactions *P*s > 0.2) (*SI Appendix*, Fig. S3*A*). Similarly, effects of 16:8 LD on sleep were stronger relative to DLE [Lighting × Time of Day interactions *F*_*Ɛ*_(2,49) = 26.74, *P* < 0.001 and *F*_*Ɛ*_(1,31) = 3.98, *P* = 0.041 for sleep proportion and duration of sleep bouts, respectively]. Under 16:8 LD, mice slept more and exhibited longer sleep bouts during ZT8 to 16 and ZT20 to 24, but they slept less and showed shorter sleep bouts during ZT0 to 4 (*SI Appendix*, Fig. S3 *B* and *C*). There was no significant difference in sleep between WT and *Opn4*^−/−^ mice (main effects of Genotype *P*s > 0.1; Lighting × Genotype interactions *P*s > 0.5; Lighting × Time of Day × Genotype interactions *P*s > 0.5). Thus, DLE exerts weaker effects on locomotor activity rhythms and sleep than a long day.

### Molecular Rhythms Are Delayed under DLE.

**Fig. 3.**
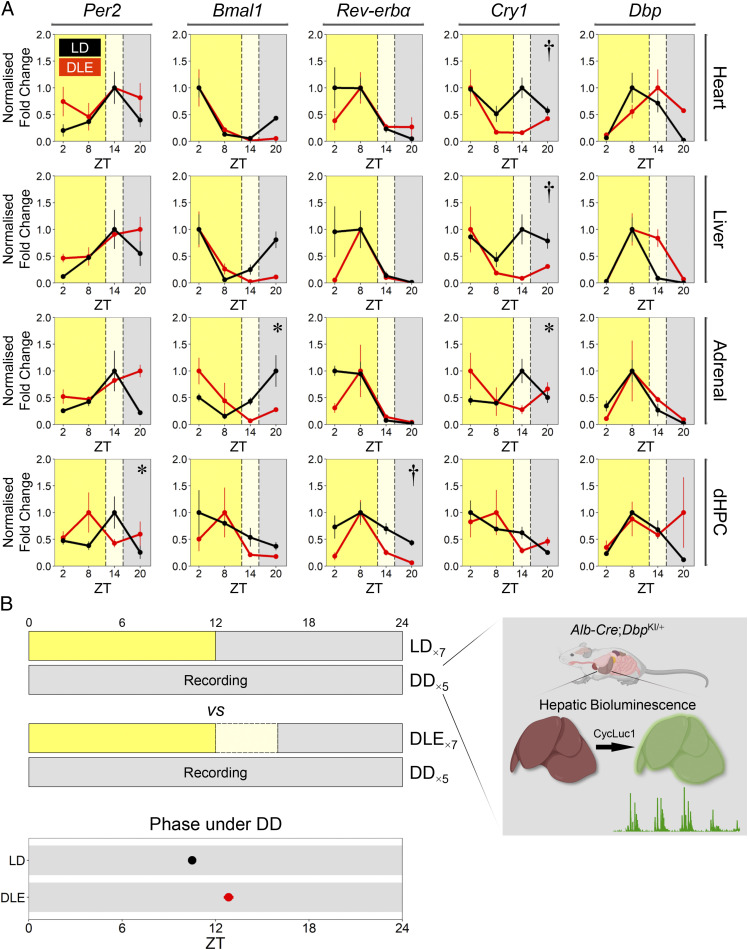
Effects of DLE on molecular clock rhythms. (*A*) Normalized fold changes in *Per2*, *Bmal1*, *Rev-erbα*, *Cry1*, and *Dbp* mRNA levels in the heart, liver, adrenal gland, and dorsal hippocampus (dHPC) at ZT2, ZT8, ZT14, and ZT20 [*n* = 4 WT mice per condition in most cases, except: *n* = 3 in the heart under LD at ZT2 (*Per2*), liver under LD at ZT2 (*Dbp*), and adrenal gland under LD at ZT8 (all target genes); *n* = 2 in the liver under LD at ZT20 (*Dbp*) and all tissues under DLE at ZT20 (all target genes)]. Data are normalized to peak expression values in each lighting condition. Under DLE, *Bmal1* and *Cry1* peak expression in the adrenal gland, as well as *Per2* peak expression in the dHPC, were delayed from the dark phase to the light phase (*Lighting × Time of Day interactions *P*s < 0.05; ^†^main effects of Lighting *P*s < 0.01). (*B*) The mean acrophase of hepatic *Dbp* bioluminescence rhythm in vivo recorded in DD from *Albumin*-*Cre*;*Dbp*^KI/+^ liver reporter mice expressing firefly luciferase in hepatocytes under the control of *Dbp* (*n* = 8 mice per Prior Lighting condition). The green trace under the illustration of the mouse liver shows 5 d of in vivo bioluminescence recording under DD from one animal. SE bars are plotted but are within the data symbols. Hepatic *Dbp* rhythm was phase delayed in mice with prior DLE exposure (main effect of Prior Lighting *P* < 0.01). Illustrations of the mouse and mouse liver are created by BioRender.

Misalignment of circadian rhythms is accompanied by changes in clock gene expression throughout the body (51). To investigate this possibility under DLE, we examined clock gene expression (*Per2*, *Bmal1*, *Rev-erbα*, *Cry1*, and *Dbp*) in the heart, liver, adrenal gland, and dorsal hippocampus at ZT2, ZT8, ZT14, and ZT20. Expression levels of target genes were normalized to the geometric mean of two housekeeping genes, *Tbp* and *Gapdh* (52); validation of these housekeeping genes is reported in *SI Appendix*, *Supplementary Methods*. Under LD, the phasing of clock gene expression observed was broadly consistent with patterns from previous mouse studies: *Per2* peaked at early night, *Bmal1* peaked near dawn, *Rev-erbα* peaked during the day, and *Dbp* peaked before the start of the night (51). Some of these molecular rhythms were delayed under DLE (Fig. 3*A*). Peaks of normalized *Bmal1* and *Cry1* messenger RNA (mRNA) levels in the adrenal gland were delayed from the dark phase to ZT2 [Lighting × Time of Day interactions *F*(3, 21) = 3.37, *P* = 0.038 and *F*(3, 21) = 3.69, *P* = 0.028, respectively]. In the dorsal hippocampus, the peak of normalized *Per2* expression was shifted from ZT14 to ZT8 [Lighting × Time of Day interaction *F*(3, 21) = 3.17, *P* = 0.046]. In addition, centers of gravity (CoG)—providing an estimate of the acrophase (φ) of the molecular rhythm (53–55)—were consistently delayed under DLE; phase shifts (∆φ) were expressed as the difference in CoG between DLE and LD (*SI Appendix*, Fig. S4). When pooled across peripheral tissues, one-sample Student’s *t* tests (two-tailed) showed that mean ∆φ values of *Rev-erbα* (2.07 ± 0.57 h) and *Dbp* (1.81 ± 0.51 h) were different from the value of 0 [one-sample *t*(3) = 3.67, *P* = 0.035 and *t*(3) = 3.55, *P* = 0.038, respectively].

The expression of six hepatic genes involved in adipogenesis and lipid/glucose metabolism (*Hmgcr*, *Hdac3*, *Ccrn4l*, *Pparγ*, *Npc1*, and *Cyp7a1*) was also examined. Notably, DLE delayed the circadian phase of the cholesterol synthesis gene *Hmgcr*: it peaked at ZT8 under LD (*SI Appendix*, Fig. S5*A*) as reported in a previous study (56); however, this was delayed to ZT14 under DLE [Lighting × Time of Day interaction *F*(3, 22) = 5.037, *P* = 0.008]. The mean ∆φ value of all six hepatic genes under investigation was 2.35 ± 0.95 h [one-sample *t*(5) = 2.49, *P* = 0.055 (two-tailed); *SI Appendix*, Fig. S5*B*].

To provide an additional functional measure of hepatic circadian output, we recorded bioluminescence signals from freely moving *Albumin*-*Cre*;*Dbp*^KI/+^ liver reporter mice, which expressed firefly luciferase in hepatocytes under the control of *Dbp* (57). Animals were kept either under LD or DLE for 1 wk; synthetic luciferin (CycLuc1) was then administered in drinking water, allowing hepatic bioluminescence emission to be detected and recorded in DD (Fig. 3*B*). Under DD, the strength of *Dbp* bioluminescence rhythm (as indicated by signal-to-noise ratios) and period length were indistinguishable between groups [main effects of Prior Lighting *F*(1, 14) = 1.84, *P* = 0.20 and *F*(1, 14) = 2.11, *P* = 0.17, respectively]. However, the acrophase of *Dbp* bioluminescence rhythm was delayed by an average of 2.34 h in mice with prior DLE exposure [main effect of Prior Lighting *F*(1, 14) = 37.13, *P* < 0.001; Fig. 3*B*]. Despite the shift in the hepatic circadian clock and metabolic rhythm, 2 wk of DLE did not affect body weight in C57BL/6 mice (*SI Appendix*, Fig. S5 *C* and *D*).

### Short-Term Memory Process Is Altered under DLE in a Sleep-Dependent Manner.

**Fig. 4.**
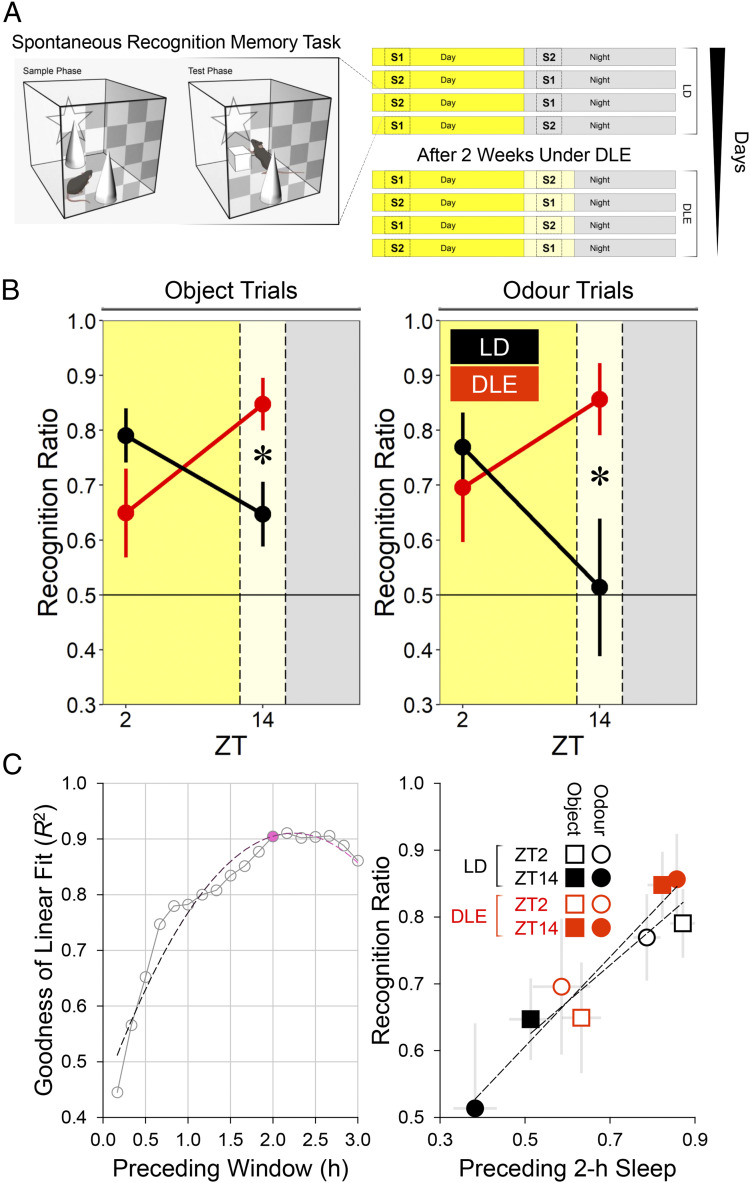
Effects of DLE on short-term memory process (*n* = 11 WT mice). (*A*) Schematic of the spontaneous recognition memory task (object trials) and summary of the order of recognition assessment in subgroup 1 (S1; 6 mice) and subgroup 2 (S2; 5 mice). The light level in the arena was 50 lx on object recognition trials and 0 lx on odor recognition trials. Illustration of the mouse in *A* is created by BioRender. (*B*) Recognition ratios from test phases of object and odor trials at ZT2 and ZT14. Higher ratios indicate stronger novelty preference, whereas ratios of 0.5 indicate no discrimination between familiar and novel stimuli. Under DLE, object and odor recognition memory performance were reversed, resulting in enhanced performance at ZT14 under DLE (*simple effect of Lighting *P* < 0.025). (*C*) Sleep proportion was determined from time windows (*t*) of varying widths in 10-min increments (*t* = 10 min, 20 min, 30 min, …, 3 h) preceding each recognition trial. (*C*, *Left*) Adopting a wider window increased the goodness of the linear fit, *R*^2^, between prior sleep and memory performance, and *R*^2^ reached an asymptotic value of ∼0.9 at *t* ∼2 h (data point in magenta). The dashed line represents the quadratic trend between *t* and *R*^2^. (*C*, *Right*) The linearity between preceding 2-h sleep and memory performance at the group level. Short and long dashed lines represent linear regression lines for object and odor trials, respectively.

Given the DLE-induced delay in circadian physiology of ∼2 h, we examined if there was any consequence for short-term memory processes at 2 h into the light phase (ZT2) and 2 h after the light phase (ZT14). We used the spontaneous recognition memory task (Fig. 4*A*) (58), which is sensitive to effects of aberrant lighting (23, 59–61). Initial analyses confirmed that under LD, there was a day/night difference in short-term object and odor recognition memory performance, with better performance at ZT2 than at ZT14 [main effect of Time of Day *F*(1, 15) = 6.59, *P* = 0.021; *SI Appendix*, Fig. S6*A*]; this is consistent with our previous findings (60). Repeated recognition assessment 2 wk later did not alter the size or direction of this effect [main effect of Repeated Testing *F*(1, 15) = 0.038, *P* = 0.85; Time of Day × Repeated Testing interaction *F*(1, 15) = 0.60, *P* = 0.45], demonstrating the stability of this behavioral rhythm under LD.

To examine the effect of DLE, another group of mice first received recognition trials at ZT2 and ZT14 under LD and at the same time points following 2 wk of DLE exposure (Fig. 4*A*). In these mice, the diurnal pattern of recognition memory performance was reversed under DLE [Lighting × Time of Day interaction *F*(1, 10) = 9.88, *P* = 0.01; Lighting × Time of Day × Type of Stimulus interaction *F*(1, 10) = 0.13, *P* = 0.73; Fig. 4*B*]. Under LD, performance was better at ZT2 than at ZT14 [simple effect of Time of Day *F*(1, 10) = 6.21, *P* = 0.032]. By contrast, under DLE performance was better at ZT14 than at ZT2 [simple effect of Time of Day *F*(1, 10) = 4.96, *P* = 0.05]. This resulted in enhanced performance at ZT14 under DLE [simple effect of Lighting *F*(1, 10) = 8.67, *P* = 0.015; Fig. 4*B*] but not at ZT2 [*F*(1, 10) = 2.45, *P* = 0.15]. The improved test performance at ZT14 was due to reduced familiar object exploration [*SI Appendix*, Fig. S6 *B*, *Right*]. In the sample phase, there was a suggestion of elevated exploratory activity at ZT2 but reduced exploration at ZT14 under DLE (*SI Appendix*, Fig. S6 *B*, *Left*). However, these effects did not reach significance [Lighting × Time of Day interaction *F*(1, 10) = 4.81, *P* = 0.053; all simple effects *P*s ≥ 0.057] and did not relate to the pattern of exploratory activity in the test phase (*SI Appendix*, Fig. S6*B*).

We then explored if the reversal of memory performance was related to changes in sleep history under DLE. To address this question, we determined the amount of sleep from time windows of varying widths (from 10 min up to 3 h in 10-min increments) preceding each recognition trial. Adopting a wider time window increased the goodness of the linear fit, *R*^2^, between prior sleep and performance, and *R*^2^ reached an asymptotic value of ∼0.9 at *t* = ∼2 h (Fig. 4 *C*, *Left*, data point in magenta). Notably, there was a strong linear relationship between preceding 2-h sleep and performance at the group level (Fig. 4 *C*, *Right*). When all individual cases were considered (11 mice × 8 trials per mouse), linear mixed-effects models (62) confirmed the benefits of preceding 2-h sleep on subsequent performance (likelihood ratio χ^2^ = 7.73, *P* = 0.005). The bootstrap 95% CI of the effect of sleep was [+0.12, +0.62], which did not overlap with zero. By contrast, sleep history did not bear any relationship to sample exploratory activity (likelihood ratio χ^2^ = 1.30, *P* = 0.25), which in turn was unrelated to test performance (likelihood ratio χ^2^ = 0.33, *P* = 0.57); 95% CIs of these effects, [−58.73, +15.02] and [−0.002, +0.001], respectively, overlapped with zero. Together, our data suggest that prolonged wakefulness at night is associated with a decline, whereas DLE-induced sleep is associated with a facilitation in short-term memory process.

### Brain cFos Signals Are Modified under DLE.

**Fig. 5.**
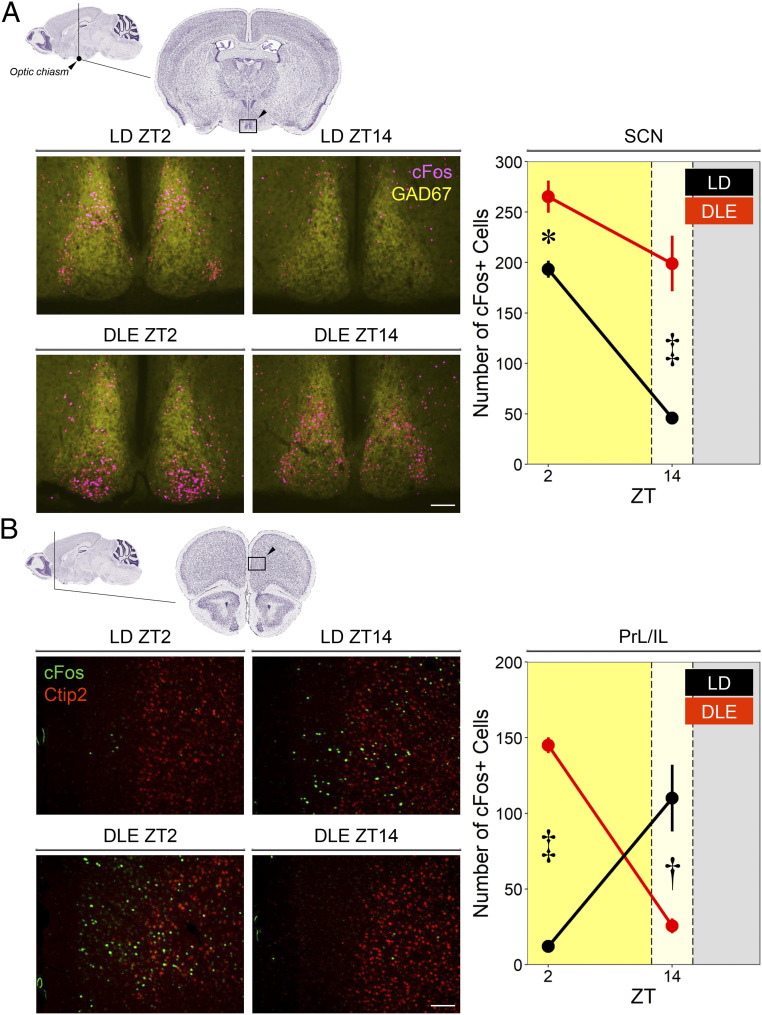
Effects of DLE on brain cFos signals (*n* = 12 WT mice; 3 mice per condition). (*A*) Representative images show immunofluorescence cFos+ cells (magenta) in the SCN, which is a heavily GAD67+ region (yellow). Coronal sections are near Bregma −0.82 mm in Franklin and Paxinos’ atlas (113), corresponding to plate 100048576_241 retrieved from the Allen Mouse Brain Atlas (114). The day/night difference in the SCN activity was dampened under DLE due to increased cFos+ cell counts (simple effects of Lighting: *P < 0.05; ^‡^*P* < 0.001). (*B*) Representative images show immunofluorescence cFos+ cells (*green*) in the prelimbic/infralimbic areas (PrL/IL) of the mPFC. Ctip2 (*red*) marks cortical layers five/six. Coronal sections are near AP Bregma +1.98 mm in Franklin and Paxinos’ atlas (113), corresponding to plate 100048576_117 retrieved from the Allen Mouse Brain Atlas (114). Cortical cFos+ cell counts were reversed under DLE (simple effects of Lighting: ^†^*P* < 0.005; ^‡^*P* < 0.001). White scale bars represent 100 μm.

To assess changes in brain states under DLE, we quantified brain immunofluorescence cFos levels in naïve mice at ZT2 and ZT14. This provides a molecular correlate of neuronal activity within the ∼1 to 2 h time window prior to learning in the recognition memory task. We found that the SCN and medial prefrontal cortex (mPFC) were differentially modified under DLE as indicated by the Lighting × Time of Day × Region interaction [*F*(1, 8) = 60.23, *P* < 0.001]. Under LD, there were more cFos+ cells in the SCN at ZT2 than at ZT14 [simple effect of Time of Day *F*(1, 8) = 39.23, *P* < 0.001], but an opposite, nocturnal pattern was observed in the mPFC [simple effect of Time of Day *F*(1, 8) = 13.74, *P* = 0.006], indicating that SCN and mPFC were out of phase under LD. Importantly, the diurnal pattern in the SCN under LD was attenuated but remained significant under DLE [simple effect of Time of Day *F*(1, 8) = 7.92, *P* = 0.023] due to increases in cFos+ cell count at ZT2 and ZT14 [simple effects of Lighting *F*(1, 8) = 9.33, *P* = 0.016 and *F*(1, 8) = 42.30, *P* < 0.001, respectively; Fig. 5*A*]. Moreover, patterns of cFos signals in the SCN dorsal and ventral subregions were dissociated under DLE (*SI Appendix*, Fig. S7), which may be suggestive of reorganization in the SCN (17–19). By contrast, the nocturnal pattern in the mPFC under LD was reversed by DLE [simple effect of Time of Day *F*(1, 8) = 75.76, *P* < 0.001]: there was an increase in the number of cFos+ cells at ZT2 but reduced signals at ZT14 [simple effects of Lighting *F*(1, 8) = 65.32, *P* < 0.001 and *F*(1, 8) = 18.74, *P* = 0.003, respectively; Fig. 5*B*]. The reversal of cFos signals was found in both superficial (layers one to four) and deep layers (layers five/six) of the mPFC (*SI Appendix*, Fig. S8).

The preoptic hypothalamus (POA) is known to regulate body cooling and promote sleep (63–67), whereas the lateral and dorsomedial hypothalamus (LHA and DMH) is known to promote wakefulness (68–70). Indeed, cFos signals in these hypothalamic regions were differentially modified by DLE as indicated by the Lighting × Time of Day × Region interaction [*F*(2, 16) = 12.47, *P* = 0.001]. In the POA, there was an increase in the number of cFos+ cells at ZT14 (*SI Appendix*, Fig. S9). By contrast, in the LHA, there was a decrease in cFos+ cell count at ZT14 (*SI Appendix*, Fig. S10 *A* and *B*). In addition, cFos+ cell counts in the LHA and DMH were positively related to each other and to the cortex but not to the POA (*SI Appendix*, Fig. S10*C*). Thus, cFos signals in the LHA/DMH versus POA were modified in a way that corresponds to their established roles in regulating wakefulness and sleep, respectively.

### Numbers of Hypothalamic and Midbrain Dopamine Cells Are Unaffected by DLE.

Exposure to long-day photoperiods for 1 to 2 wk is known to decrease the number of dopamine tyrosine-hydroxylase–expressing (TH+) cells in the paraventricular nuclei of the hypothalamus (PVN), increasing behavioral immobility and reducing exploratory activity in rats and mice (20, 21). Similarly, seasonal variation in photoperiod is associated with changes in TH levels in the human midbrain (22). To assess this possibility under DLE, we examined immunofluorescence TH levels in the PVN as well as in the ventral tegmental area (VTA) and substantia nigra pars compacta (SNc), which provide major dopaminergic projections to different parts of the brain. DLE did not reduce the number of TH+ cells in the PVN [main effect of Lighting *F*(1, 8) = 0.51, *P* = 0.50; Lighting × Time of Day interaction *F*(1, 8) = 1.08, *P* = 0.33; *SI Appendix*, Fig. S11, *Upper*] or in the VTA/SNc [main effect of Lighting *F*(1, 8) = 0.82, *P* = 0.39; Lighting × Time of Day interaction *F*(1, 8) = 0.24, *P* = 0.64; *SI Appendix*, Fig. S11, *Lower*].

## Discussion

A total of 2 wk of 4-h, 20-lux DLE exerts widespread effects on circadian physiology and behavior in C57BL/6 mice; it 1) delays locomotor activity onsets, midpoints, and offsets (Fig. 1); 2) promotes sleep and body cooling, postponing the accumulation of sleep pressure in the next morning (Fig. 2 and *SI Appendix*, Fig. S2); 3) delays molecular clock rhythms in peripheral tissues (Fig. 3); 4) reverses the diurnal pattern in short-term object and odor recognition memory performance, which is associated with changes in sleep history (Fig. 4); and 5) modifies patterns of hypothalamic and cortical cFos signals (Fig. 5). DLE-induced phase delays are unaffected in *Opn4*^−/−^ mice, indicating that rods and cones are capable of driving these responses in the absence of melanopsin as has been described for other circadian responses (42–44). However, these data do not preclude a contribution of melanopsin to these responses under normal conditions. It could be argued that DLE-induced phase delays are simply the result of negative masking (i.e., suppression of locomotor activity by dim light) (71). However, this cannot explain phase delays in locomotor activity offset and *T*_skin_ offset, both of which should have been masked by the start of the light phase. Moreover, the persistence of DLE-induced phase changes in locomotor activity and hepatic *Dbp* bioluminescence rhythm under DD indicates that these effects are not the result of negative masking per se.

There are similarities between 20-lux DLE and a long-day photoperiod. Under both conditions, locomotor activity onsets and midpoints are phase shifted, and object recognition memory performance is improved relative to 12:12 LD (23). However, there are also key differences. We do not see a compression of locomotor activity into the dark phase, which is observed under long-day photoperiods (16). And unlike long days (20, 21), 20-lux DLE does not reduce the number of dopamine TH+ cells in the hypothalamus or midbrain. Moreover, in contrast to DLAN (27–30, 32–35), 4-h DLE does not affect body weight despite delaying hepatic circadian rhythms and clock outputs. Thus, the effects of DLE in mice appear to be milder than either long photoperiods or DLAN. A comparison of these paradigms is summarized in *SI Appendix*, Table S2. However, it should be noted that the effects of long photoperiods on TH reduction in previous studies have used more extreme 19:5 LD conditions (20, 21); as such, longer duration DLE exposure (7 h) may exert comparable effects. It also remains to be determined if longer periods of exposure to DLE (>2 wk) will have more severe consequences for brain dopamine signaling or metabolic processes.

Under DLE, circadian rhythms, sleep patterns, and short-term memory processes are realigned, adopting a delayed phase relationship with the extended day. Such coordinated realignment of circadian physiology and behavior is as an adaptive response to environmental changes crucial for survival (19, 72, 73). In fact, the dissociation of SCN subregional cFos signals under DLE (*SI Appendix*, Fig. S7) is suggestive of some plasticity because weakened dorsal–ventral SCN coupling can maximize adaptability of the SCN to the extended day (17–19). This effect needs to be confirmed in future studies using more direct readouts of neuronal activity (17). Under more severe aberrant lighting protocols, such as T7 (3.5-h day/3.5-h night) (59, 61), T20 (10-h day/10-h night) (74, 75), and jet lag (76, 77), sleep and circadian processes fail to adopt a stable phase relationship with the environment. Maladaptation of the circadian system perturbs cardiovascular, metabolic, and memory functions (27–30, 32–35, 51, 59, 61, 74, 76, 77). By contrast, DLE has milder effects: it delays the hepatic circadian rhythms and clock outputs without affecting body weight, and it reverses the day/night pattern of short-term memory performance without causing any behavioral impairment.

While the 20-lux DLE used in this study was based upon human artificial light exposure (14, 15, 36, 37), this is likely to be relatively brighter for mice. For example, the half-maximal effective light intensity (EC_50_) for human phase-shifting responses to 6.5-h light varies between 12.54 (460 nm) to 13.07 (555 nm) log quanta (78). However, in mice phase-shifting responses to a 15-min light stimulus have an EC_50_ of 11.41 log quanta (6); this is more than a log unit lower despite the 3-log-unit-shorter stimulus duration. Certainly, due to differences in the geometry of the human versus rodent eye, the 20-lux DLE will result in greater effective retinal irradiance in mice in comparison with humans. As a result, the light level used in this study may be relatively brighter for mice than for humans.

Despite having a higher sensitivity to light, circadian responses of mice are not qualitatively different from those in humans. In both species, light presented during the early biological night delays circadian rhythms, whereas light presented later at night causes phase advance (5, 38, 39). This is evident from our results: the 4-h, 20-lux DLE induces a comparable phase delay of ∼2 to 3 h in our mice (Fig. 1) as has been observed in humans (14, 15, 36). The resemblance seems to be at odds with our recent review of the literature, which suggests that humans require brighter (>100 lx) and longer (>30 min) light for photoentrainment, whereas mice can entrain to dimmer (<1 lx) and shorter (15 min) light (5); this difference may partly reflect how visual systems of diurnal and nocturnal species evolved and adapted to their distinct ecological niche (5). However, procedural differences between human and mouse studies may also be important. For example, human studies often adopt the constant routine protocol, which involves keeping participants under hours of dim ambient lighting (<10 lx) prior to the presentation of the phase-shifting light stimulus (79); thus, the participant’s visual system is adapted to dim light beforehand. By contrast, mouse studies usually present phase-shifting light stimuli in total darkness (39), which may evoke a greater relative response. Some support for this hypothesis comes from human studies in which light history has been shown to directly affect the magnitude of circadian responses to light (80, 81). Taken together, a direct comparison of nonvisual responses to light between humans and mice is not straightforward, and differences in protocols and other extraneous factors must also be considered.

Another potentially relevant difference is the C57BL/6 mouse’s deficiency in pineal melatonin synthesis (82, 83), which is associated with sleep onset at the start of the biological night in humans (84, 85). Nevertheless, despite the lack of pineal melatonin, the PRC of the C57BL/6 mouse is similar to the human PRC (5, 38, 39). Furthermore, in a previous study comparing melatonin-proficient versus melatonin-deficient mice, these animals were statistically indistinguishable in terms of entrainment to skeleton photoperiods, free-running period length, acute suppression of wheel-running activity by light, and light-induced phase shift (86). While we cannot exclude the possibility of subtle effects of melatonin on circadian responses to light, all available data suggest that the lack of pineal melatonin in C57BL/6 mice will not overtly affect our results.

Sleep‒wake cycles are regulated by circadian and homeostatic processes (87). Although our results can be explained in terms of a change in phasing of the internal clock (process *C*), they are equally compatible with an account based on sleep homeostasis (process *S*); it could be argued that DLE promotes sleep, postponing the accumulation of sleep pressure in the next morning, without affecting the phase of the internal clock. While our data are compatible with a change in either process *C* or *S*, in reality, these processes are intricately related (88). For example, adenosine-dependent changes in sleep pressure can affect the SCN (89). On the other hand, the SCN can exert an influence on sleep-regulatory and thermoregulatory neurons in the preoptic hypothalamus via a) monosynaptic projections to the medial preoptic area, which projects to the ventrolateral preoptic nucleus (VLPO), or b) multisynaptic pathways involving the subparaventricular zone (SPVZ) and dorsomedial hypothalamic nucleus (DMH) (90–93). Furthermore, in *Cry1*,*2*^−/−^ mice lacking process *C*, their process *S* is also compromised (94). Thus, it is likely that processes *C* and *S* are both affected under DLE; separating the contributions of these equally important processes may prove challenging (88).

In this regard, both processes *C* and *S* are important determinants of behavioral performance. The reversed pattern of performance under DLE could partly be related to the phase shift in the hippocampal molecular clock (Fig. 3), as the hippocampus is involved in some aspects of recognition memory processes (95, 96), and the level of hippocampal *Per1*,*2* is related to performance (97, 98). In addition to circadian processes, sleep history could be crucial (Fig. 4*C*), potentially facilitating the recovery of attentional processes required for learning (99, 100); this notion is widely appreciated based on numerous human studies (1, 101–103) but has not been thoroughly investigated in animal models (100). The role of DLE-induced sleep in restoring attentional processes remains to be examined in other behavioral tasks (e.g., the five-choice serial reaction time task and psychomotor vigilance task).

In summary, we demonstrate that DLE causes coordinated realignment of circadian rhythms, sleep patterns, and short-term memory processes in mice, which is likely to be experienced by the majority of the populace on a daily basis. Our results complement and extend the findings from nonlaboratory studies in humans, highlighting the biological impact of DLE and emphasizing the need to optimize our lighting regimes and promote circadian health.

## Methods

### Animals and Housing Conditions.

A total of 111 mice were used in this study. In experiments examining home cage activity and sleep, there were 12 WT mice of C57BL/6 background (*Opn4*^+/+^) and 12 *Opn4*^−/−^ mice (equal numbers of ♀ and ♂) obtained from crossbreeding heterozygous *Opn4*^+/−^ mice (104). Detailed analyses of locomotor activity and immobility-defined sleep under normal and abnormal lighting conditions did not reveal any difference between sexes or genotypes; thus, male C57BL/6 WT mice (Envigo; RRID: IMSR_JAX:000664) were used in subsequent experiments. In the DD experiment in which in vivo hepatic bioluminescence and locomotor activity were examined, 16 *Albumin*-*Cre*;*Dbp*^KI/+^ liver reporter mice (9♀ and 7♂) were used (57). All mice were singly housed with ad libitum access to food and water. Cages were placed within light-tight ventilated chambers (LTC) equipped with multiple cool-white light-emitting diodes (LEDs) (Luxeon Star LEDs; Quadica Developments), providing a light level of 200 lx during the day; the spectral power distribution of the LEDs consisted of a higher and narrower peak at ∼460 nm and a lower and broader peak at ∼560 nm. Each LTC was also equipped with PIR for long-term recording of locomotor activity (49). Animals were kept in LTC under 12-h day/12-h night cycles (12:12 LD) for at least 2 wk, before receiving dim light (20 lx) in the evening from ZT12 to 16 (DLE) or 16-h day/8-h night cycles (16:8 LD). The temperature of the animal holding room was maintained at 19 to 21 °C. Experimental procedures involving C57BL/6 WT and *Opn4*^−/−^ mice were conducted at the University of Oxford, England in accordance with the United Kingdom Animals (Scientific Procedures) Act 1986 under Project License PE4ED9D2C/PP0911346 and Personal Licenses I869292DB and IDB24291F. The DD experiment involving *Albumin*-*Cre*;*Dbp*^KI/+^ mice was conducted at Smith College, Massachusetts in accordance with the standard of the Association for Assessment and Accreditation of Laboratory Animal Care International.

### Locomotor Activity Phase Markers.

Raw PIR data with a temporal resolution of 10 s were first smoothed with a 1-h moving average across the entire recording period. Following ref. 105, activity onsets and offsets were defined as the intersections between a short moving average (3-h window) and a long moving average (24-h window) of the smoothed time series. More specifically, for each time bin (*i*), the difference between short and long moving averages (Δ_*i*_) was determined, and the product of Δ_*i*−1_ and Δ_*i*_ was calculated. The daily onset of activity was the time point at which the short moving average first exceeded the long moving average, that is, when Δ_*i*−1_Δ_*i*_ < 0 and Δ_*i*−1_ < 0, whereas daily activity offsets were the time points at which the short moving average dropped below the long moving average, that is, when Δ_*i*−1_Δ_*i*_ < 0 and Δ_*i*−1_ > 0. Activity midpoints were defined as the time points at which total activity in the preceding 8 h was the same as total activity in the subsequent 8 h (105). To implement this, we determined the difference between preceding 8-h activity and subsequent 8-h activity (Δ_*i*_) for each time bin and calculated the product of Δ_*i*−1_ and Δ_*i*_. Activity midpoints were estimated as the time points at which Δ_*i*−1_Δ_*i*_ < 0 and Δ_*i*−1_ < 0. Activity onset, midpoint, and offset calculations were conducted in R (RRID: SCR_001905).

### Clock Gene and Metabolic Gene Expression.

#### Tissues collection.

Mice were housed under LD for 4 wk; half of the animals were then exposed to DLE, while the other half remained under LD. After 2 wk of exposure to DLE, animals were killed by cervical dislocation at ZT2, ZT8, ZT14, or ZT20. Samples from the heart, liver, adrenal gland, and dorsal hippocampus (including dentate gyrus, CA1, and CA3) were collected and flash frozen on dry ice before being stored at −80 °C.

#### RNA extraction.

Tissue samples were homogenized in 500 μL TRIzol (Thermo Fisher Scientific) and incubated at room temperature for 5 min before adding 100 μL chloroform (Sigma Aldrich) and spinning for 15 min at 12,000 RPM at 4 °C. After phase separation, the clear lysate containing RNA was removed and further purified using the RNeasy Plus Mini Kit (Qiagen). RNA was quantified using a Nanodrop1000 (Thermo Fisher Scientific).

#### RT-qPCR.

Complementary DNA (cDNA) was synthesized from extracted RNA using SuperScript VILO Master Mix (Thermo Fisher Scientific), adding 250 ng RNA to the reaction where possible. RT-qPCR of 11 genes of interest (*Per2*, *Bmal1*, *Rev-erbα*, *Cry1*, *Dbp*, *Hmgcr*, *Hdac3*, *Ccrn4l*, *Pparγ*, *Npc1*, and *Cyp7a1*) was performed using TaqMan Gene Expression Assays (Thermo Fisher Scientific) by the StepOnePlus Real-Time PCR System (Applied Biosystems); for the two housekeeping genes (*Tbp* and *Gapdh*), RT-qPCR was performed using Fast SYBR Green (Thermo Fisher Scientific). TaqMan Gene Expression Assays (Thermo Fisher Scientific) for the genes of interest were mm00478099_ml (*Per2*), mm00500223_ml (*Bmal1*), mm00520708_ml (*Rev-erbα*), mm00514392_ml (*Cry1*), mm00497539_ml (*Dbp*), mm01282499_m1 (*Hmgcr*), mm00515916_m1 (*Hdac3*), mm00802276_m1 (*Ccrn4l*), mm00440940_m1 (*Pparγ*), mm00435300_m1 (*Npc1*), and mm00484152_m1 (*Cyp7a1*). The primer sequences 5′ to 3′ for *Tbp* were TGG​GCT​TCC​CAG​CTA​AGT​TC (forward) and GGA​AAT​AAT​TCT​GGC​TCA​TAG​CTA​CTG (reverse), and for *Gapdh*, they were TGC​ACC​ACC​AAC​TGC​TTA​G (forward) and GATGCAGGGATGATGTTC (reverse). During RT-qPCR, each 96-well plate was loaded with cDNA samples from all four time points from each lighting condition; cDNA samples from LD and DLE conditions were amplified on separate plates.

#### Quantification of gene expression and phase shifts.

mRNA levels of the genes of interest were quantified using the comparative threshold cycle (C_T_) method (106). The 2^−*C*T^ values of these genes were expressed as a ratio relative to the geometric mean of the 2^−*C*T^ values of the housekeeping genes in the same sample (2^−Δ*C*T^); validation of housekeeping genes is reported in *SI Appendix*, *Supplementary Methods*. Individual 2^−Δ*C*T^ values were then divided by the highest mean 2^−Δ*C*T^ value in each lighting condition to obtain 2^−ΔΔ*C*T^, indicating changes in mRNA level relative to maximal expression. In addition, CoG were obtained from the CircWave software (53–55). These values provided an estimate of the acrophase (φ) of the molecular rhythm under LD and DLE (φ_LD_ and φ_DLE_, respectively). The extent of the phase shift, Δφ, was calculated from φ_DLE_ − φ_LD_.

### In Vivo Hepatic Dbp Bioluminescence.

A total of 16 *Albumin*-*Cre*;*Dbp*^KI/+^ mice expressing firefly luciferase in hepatocytes under the control of *Dbp* were used to measure hepatic *Dbp* bioluminescence signals in vivo (57). They were first singly housed under LD for at least 8 wk. Half of them were exposed to DLE for a week, whereas the other half remained under LD. Synthetic luciferin CycLuc1 (Tocris Bioscience) was then administered by dissolving CycLuc1 in dimethyl sulfoxide (DMSO) and diluting the 50 mM stock solution to 0.1 mM in drinking water. The animal’s back and abdominal hair was shaved to optimize detection of hepatic bioluminescence under DD. The mouse cage was placed inside the dark LumiCycle In Vivo recording unit (Actimetrics) equipped with two photomultiplier tubes above the cage for detecting bioluminescence emission. A programmable shutter covered the photomultiplier tubes periodically (1 min in every 15 min) to record background counts and obtain background-corrected bioluminescence counts every minute. Hepatic bioluminescence was recorded in DD for 5 d. Discrete wavelet transform was conducted on bioluminescence data as described previously (107, 108). Locomotor activity under DD was recorded from PIR (K-940; Visonic); activity onset, midpoint, and offset were determined as described in *Locomotor Activity Phase Markers*.

### Spontaneous Object and Odor Recognition.

Recognition testing took place in a 25 × 25 × 25 cm transparent acrylic arena with two wallpapers attached to the exterior walls. One wallpaper was a chequerboard pattern with 4 × 4 cm black and white squares. The wallpaper on the opposite side was black with a white symmetrical five-point star shape of 14 × 14 cm. An infrared camera and a white LED light were positioned above the center of the arena. The light level was 50 lx on object trials and 0 lx on odor trials. *SI Appendix*, Table S3 provides a description of all eight different objects and eight odors used in the experiment. There were multiple replicates of each object, and different replicates of the same object were presented in sample and test phases. The arena and objects were wiped with 70% ethanol after each phase.

Mice received 10-min acclimatization trials in the empty arena at ZT8 (midway between ZT2 and ZT14) for 5 d. They then received object and odor recognition trials at ZT2 and ZT14 and at the same ZTs after 2 wk of exposure to DLE. During the sample phase of each trial, two identical replicates of an object or an odor cue were placed in the arena; the sample phase was terminated after 10 min. After a 3-min delay, novelty preference was assessed in the arena, now containing a third replicate of the familiar sample and a novel stimulus; the test phase was terminated after 3 min. For six of the animals (subgroup *1*), the order of recognition trials under LD was the following: ZT2 (*odor*), ZT14 (*odor*), ZT14 (*object*), and ZT2 (*object*); whereas for the remaining animals (subgroup *2*), the arrangement was reversed: ZT14 (*odor*), ZT2 (*odor*), ZT2 (*object*), and ZT14 (*object*). Under DLE, the order of assessment was ZT2 (*object*), ZT14 (*object*), ZT2 (*odor*), and ZT14 (*odor*) for subgroup *1*; for subgroup *2*, the order was ZT14 (*object*), ZT2 (*object*), ZT14 (*odor*), and ZT2 (*odor*). The order of recognition assessment is summarized in Fig. 4*A*. The identity of novel and familiar stimuli and their spatial positions in the arena at test were counterbalanced in order to take into account any potential stimulus-specific or location-specific bias. Automated tracking of exploratory activity was conducted in ANY-maze (Stoelting; RRID:SCR_014289), which tracked the position of the mouse's snout every 0.1 s and recorded stimulus exploration times. Test performance was expressed as the proportion of time spent with the novel stimulus. Rodents show the strongest novelty preference within the first 30 s of stimulus exploration (95). This declines rapidly over time and reaches chance level by the second and third minutes of testing (109, 110). Thus, our main analyses focused on 0 to 60 s of test performance; data from 60 to 120 s and 120 to 180 s of the test phase are summarized in *SI Appendix*, Table S4. As sample exploratory activity levels were >25 s across the four lighting/ZT conditions (range = 25.25 to 130.60 s when pooled across object and odor trials), with average exploration times (*SI Appendix*, Fig. S6 *B*, *Left*) similar to previous mouse studies (111, 112), no post hoc or arbitrarily defined threshold was used to exclude any data point from analyses.

### Brain cFos Signals.

Mice housed under LD or DLE were injected intraperitoneally with sodium pentobarbitone at ZT2 or ZT14. Immediately after the loss of the pedal reflex, they were perfused transcardially with phosphate-buffered saline (PBS) and 4% paraformaldehyde (PFA). The brain was extracted from the skull, kept in 4% PFA overnight, rinsed with PBS the next day, and transferred into 30% sucrose solution for cryopreservation. After 2 to 3 d, brains were sectioned coronally at 50 μm on a freezing microtome (Leica). Based on Franklin and Paxinos’ atlas (113), coronal sections near Bregma +1.98, +0.14, −0.82, and −1.94 mm were selected to examine cFos signals in the mPFC, POA, SCN, and LHA/DMH, respectively; these correspond to plates 100048576_117, 100048576_209, 100048576_241, and 100048576_281 retrieved from the Allen Mouse Brain Atlas (114). The selected sections for each region from all four conditions were processed on the same days to minimize variability in staining quality. Sections were first washed in PBS, blocked in normal donkey serum (Jackson ImmunoResearch; RRID: AB_2337258) for at least 90 min, and incubated at 4 °C with rabbit anti-cFos (dilution 1:4,000; Synaptic Systems; RRID: AB_2619946), a molecular correlate of recent neuronal activity. In addition, for mPFC sections, the rat anti-Ctip2 antibody (dilution 1:250; Abcam; RRID: AB_2064130) was added to serve as a marker for deep cortical layers (layers five/six); for SCN sections, the mouse anti-GAD67 antibody (dilution 1:500; Merck Millipore; RRID: AB_2278725) was added to serve as a marker for the SCN; and for other hypothalamic sections, the chicken anti-TH antibody (dilution 1:500; Abcam; RRID: AB_1524535) was added to mark hypothalamic TH-immunoreactive cell bodies and fibers. After 72 h of primary antibody incubation, POA, SCN, and LHA/DMH sections were washed in PBS and incubated at room temperature with Alexa Fluor 488 donkey anti-chicken, Cy3 donkey anti-rabbit, and Alexa Fluor 647 donkey anti-mouse secondary antibodies (dilution 1:500; Jackson ImmunoResearch; RRID: AB_2340375, RRID: AB_2307443, and RRID:AB_2340863, respectively), whereas mPFC sections were incubated with Alexa Fluor 488 donkey anti-rabbit and Cy3 donkey anti-rat secondary antibodies (Jackson ImmunoResearch; RRID: AB_2313584 and RRID: AB_2340667, respectively). After 4 h of secondary antibody incubation, sections were washed in PBS. Finally, stained sections were mounted on glass slides and coverslipped with Vectashield (Vector Laboratories; RRID: AB_2336789). Processed sections were scanned using a confocal microscope (Fluoview FV1000; Olympus) under 10× and 20× objective lenses. Identical settings were used to acquire images of each region from the four different conditions, and cFos-immunoreactive nuclei were quantified in Fiji ImageJ (115).

### Statistical Analyses.

Means ± SEs of the mean were plotted in figures, and statistical testing was conducted in Statistical Package for the Social Sciences (SPSS) (IBM; RRID: SCR_002865) and R (RRID: SCR_001905). α = 0.05 was adopted in all analyses unless otherwise specified. To analyze behavioral data (locomotor activity phases, immobility-defined sleep, recognition memory performance, and stimulus exploration times), we conducted within-subjects and split-plot ANOVAs with Lighting, Time of Day, and Type of Stimulus as within-subjects factors and Sex and Genotype as between-subjects factors; for locomotor activity under DD, we conducted between-subjects ANOVAs with Prior Lighting as a factor. To analyze molecular data (clock gene expression and cFos+ cell counts), Lighting × Time of Day between-subjects ANOVAs and Lighting × Time of Day × Region split-plot ANOVAs were conducted. Significant interaction effects in most cases were followed up with simple effect analyses in SPSS (116). For main and interaction effects involving within-subjects factors with more than two levels, Greenhouse–Geisser corrections were applied to the degrees of freedom (*df*) whenever the assumption of sphericity was violated (117); these *F* values with adjusted *df* were denoted as *F*_*Ɛ*_. To examine the effects of sleep history and sample exploratory activity on test performance, we conducted linear mixed-effects models in R (62), with preceding 2-h sleep proportion or sample exploratory activity as the fixed effect, individual mice as the random effect, and recognition scores as the response variable. The significance of the fixed effect was assessed by examining the change in deviance (−2 × maximum log-likelihood) from comparing linear mixed-effects models with versus without the fixed effect of interest. A significant drop in deviance—determined by the likelihood ratio χ^2^ value—indicates that incorporating the fixed effect improves model fitting and explains more variance in the response variable than when it is excluded from the model (118). The 95% CI of the fixed effect (slope) was computed from parametric bootstrap with 10,000 iterations.

## Supplementary Material

Supplementary File

## Data Availability

Data reported in this manuscript were deposited by S.K.E.T. on Figshare (September 8, 2021) and can be accessed via https://figshare.com/articles/dataset/Tam_etal_data_xlsx/16583834. Supplementary methods, tables S1 to S7, and figures S1 to S11 can be found in the *SI Appendix*.
