# Supplementary material for: Dim light in the evening causes coordinated realignment of circadian rhythms, sleep, and short-term memory
Source: Proc Natl Acad Sci U S A. 2021 Sep 23;118(39):e2101591118. doi: 10.1073/pnas.2101591118 (PMC8488663; doi:10.1073/pnas.2101591118)
Supplement: Supplementary File [file pnas.2101591118.sapp.pdf]

PNAS
www.pnas.org

Supplementary Information for

Dim Light in the Evening Causes Coordinated Realignment of Circadian Rhythms, Sleep, and Short-term Memory

Shu K. E. Tam, Laurence A. Brown, Tatiana S. Wilson, Selma Tir, Angus S. Fisk, Carina A. Pothecary, Vincent van der Vinne, Russell G. Foster, Vladyslav V. Vyazovskiy, David M. Bannerman, Mary E. Harrington, and Stuart N. Peirson*

***Correspondence:** Stuart N. Peirson
Email: stuart.peirson@eye.ox.ac.uk

This PDF file includes:

Supplementary Methods
Supplementary Tables S1–S7
Supplementary Figures S1–S11
Supplementary References

SUPPLEMENTARY METHODS

Validation of Housekeeping Genes

Changes in gene expression between groups may be due to differences in RNA loading between samples (reflecting differences in RNA quantity, quality, or reverse transcription efficiency) as well as diurnal changes in expression. To account for differences in RNA loading, we initially used three different housekeeping genes (*Arbp*, *Tbp*, and *Gapdh*) to normalise expression of target genes of interest. The final choice of housekeeping genes was based upon the Gene Stability Index (GSI), a measurement of the variability of housekeeping genes when compared against each other across all samples and conditions; a low value indicates a gene with a stable pattern of expression across all samples [1]. Mean GSI values for *Arbp*, *Gapdh*, and *Tbp* were 2.32, 2.05, and 1.90, respectively (**Table S5**). *Tbp* and *Gapdh* were the most stable housekeeping genes across all samples, and as such, the geometric mean of *Tbp* and *Gapdh* was used as a normalisation factor [1]. In addition, for each housekeeping gene effects of Time of Day were examined for each tissue/lighting condition using one-way ANOVAs (**Table S6**). Among these conditions, *Tbp* showed a significant difference over time in the liver (LD) and hippocampus (LD), whereas *Gapdh* showed a significant difference over time in the heart (DLE), adrenal gland (DLE), and hippocampus (LD). Whilst *Arbp* showed no significant differences across any tissue or condition, this was due to the high variance of *Arbp* within groups (illustrated by relatively high GSI values in **Table S5**). A normalisation factor based upon the geometric mean of the two most stable genes (*Tbp* and *Gapdh*) did not show significant differences over time, except in the hippocampus (LD). On closer inspection, this difference was found to be due to higher expression of all 3 housekeeping genes in one group (ZT20). As such, this is more likely to reflect a higher RNA loading in this group rather than a diurnal variation in housekeeping expression. Finally, to confirm the accuracy of clock gene expression patterns reported in this study, we also compared our data under control conditions (LD) with previously published work. The acrophase of clock gene expression was broadly consistent with previously published data under LD from seven independent studies (**Table S7**).

SUPPLEMENTARY TABLES S1–S7

Table S1. Immobility-defined Sleep in Hours (Mean ± Standard Error of the Mean) Under LD and DLE

Table S2. DLE Effects are Milder Than Effects of Other Lighting Protocols in Previous Mouse Studies

Table S3. Objects and Odour Stimuli Used in the Spontaneous Recognition Memory Task

Table S4. Mean Recognition Scores (± Standard Error of the Mean) Across the Three Minutes of the Test Phase

Table S5. Gene Stability Indices for Housekeeping Genes *Arbp*, *Tbp*, and *Gapdh*

Table S6. *P*-values from One-way ANOVAs Examining Effects of Time of Day on Housekeeping Gene Expression

Table S7. Acrophase of Clock Gene Expression Under LD: A Comparison Between Previously Published Studies and the Current Study

Table S1. Immobility-defined Sleep in Hours (Mean ± Standard Error of the Mean) Under LD and DLE

GENOTYPE	LIGHTING	DAY			NIGHT			24-h TOTAL
		ZT00–04	ZT04–08	ZT08–12	ZT12–16	ZT16–20	ZT20–24	
WT	LD	3.13 ± .11	3.60 ± .05	3.41 ± .09	.79 ± .15	1.84 ± .20	2.55 ± .16	15.31 ± .59
	DLE	2.54 ± .15	3.51 ± .06	3.69 ± .05	2.20 ± .20	.99 ± .20	2.21 ± .21	15.15 ± .58
Opn4$^{-/-}$	LD	2.94 ± .14	3.57 ± .04	3.23 ± .09	.78 ± .11	1.51 ± .17	2.48 ± .11	14.51 ± .38
	DLE	2.29 ± .21	3.54 ± .04	3.64 ± .03	1.97 ± .11	.87 ± .17	2.41 ± .14	14.73 ± .41
WT 12-h TOTAL	LD	10.14 ± .21			5.17 ± .43			
	DLE	9.74 ± .21			5.40 ± .41			
Opn4$^{-/-}$ 12-h TOTAL	LD	9.74 ± .16			4.77 ± .32			
	DLE	9.47 ± .23			5.26 ± .25			

Note—No significant difference in total 12-h or 24-h sleep between LD and DLE or between genotypes (all *ps* > .05).

Table S2. DLE Effects are Milder Than Effects of Other Lighting Protocols in Previous Mouse Studies

MEASURE	LIGHTING		
	DLE*	LONG-DAY PHOTOPERIOD	DIM LIGHT FOR ENTIRE NIGHT‡
1. Rest–activity rhythm	Delayed	Delayed*	Advanced T_{core} [*e*]
2. Clock gene expression	Delayed	Dampened [*a*]	Dampened [*f*]
3. Short/long-term memory	Improved	Improved [*a*]	Slightly reduced ^{NS} [*g*]
4. Body weight	*NS*	Slightly increased ^{NS} [*b*,*c*]†	Increased [*e*,*f*,*g*,*h*]
5. Liver metabolism	Delayed		Disrupted [*e*,*h*]
6. Hypothalamic dopamine	*NS*	Decreased [*d*]	

Note—[*a*] Dellapolla, A. *et al*. (2017). *Scientific reports*, *7*(1), 3925; [*b*] Nelson, R. J. (1990). *Physiology & behavior*, *48*(3), 403–408; [*c*] Kawai, H. *et al*. (2018). *Biomedical research*, *39*(1), 47–55; [*d*] Young, J. W. *et al*. (2018). *Neuropsychopharmacology*, *43*(8), 1721–1731; [*e*] Borniger, J. C. *et al*. (2014). *Chronobiology international*, *31*(8), 917–925; [*f*] Fonken, L. K. *et al*. (2013). *Journal of biological rhythms*, *28*(4), 262–271; [*g*] Fonken, L. K., & Nelson, R. J. (2013). *Behavioural brain research*, *243*, 74–78; [*h*] Fonken, L. K. *et al*. (2010). *Proceedings of the National Academy of Sciences of the United States of America*, *107*(43), 18664–18669. *Results from the current study; †In study [*b*], the effect of a long day was compared with a short day; ‡In studies [*e*], [*f*], [*g*], and [*h*], the effect of dim light for the entire night was compared with a long day rather than 12:12 LD. ^{NS} = statistically-nonsignificant effects; T_{core} = core body temperature.

Table S3. Objects and Odour Stimuli Used in the Spontaneous Recognition Memory Task

TRIAL	LIGHTING	SUBGROUP*/ ZT	STIMULUS PAIR†
Object	LD	Subgroup *1* at ZT14	***A***—Clear glass candle holder with a circular opening, length 4.5 cm × width 4.5 cm × height 3 cm; opening 2.2 cm diameter × depth 1.8 cm
		Subgroup *2* at ZT02	***B***—Clear glass pyramid paperweight with a dark blue glass base, length 5.1 cm × width 5.1 cm × height 8 cm
		Subgroup *1* at ZT02	***C***—Silver-coloured plastic spherical ornament, diameter 5.3 cm
		Subgroup *2* at ZT14	***D***—Brown plastic bottle with yellow labels and a green metal lid, base diameter 3.4 cm × height 8.7 cm
	DLE	Subgroup *1* at ZT02	***E***—Clear glass jar (no lid), base diameter 4.3 cm × height 8 cm
		Subgroup *2* at ZT14	***F***—Wood block with a red triangular top, length 3.5 cm × width 1.7 cm × height 3.5 cm
		Subgroup *1* at ZT14	***G***—LED spotlight bulb with a glass silver-coloured base and a white plastic top, base diameter 4.8 cm × height 5.9 cm
		Subgroup *2* at ZT02	***H***—Green flower-shaped Lego Duplo attached to a yellow Lego Duplo square block, length 4.1 cm × width 4.1 cm × height 4.6 cm
Odour‡	LD	Subgroup *1* at ZT02	***I***—0.5 mL purple yam flavouring (McCormick, Quezon City, Philippines) in a brown glass vial
		Subgroup *2* at ZT14	***J***—0.5 mL coconut flavouring (Waitrose, Bracknell, England) in a brown glass vial
		Subgroup *1* at ZT14	***K***—0.5 mL chocolate flavouring (Uncle Roy's Comestible Concoctions, Ayr, Scotland) in a brown glass vial
		Subgroup *2* at ZT02	***L***—0.5 mL butterscotch flavouring (Uncle Roy's Comestible Concoctions, Ayr, Scotland) in a brown glass vial
	DLE	Subgroup *1* at ZT02	***M***—0.5 mL mango flavouring (Stef's Baking Supplies, Ayr, Scotland) in a brown glass vial
		Subgroup *2* at ZT14	***N***—0.5 mL apple flavouring (Uncle Roy's Comestible Concoctions, Ayr, Scotland) in a brown glass vial
		Subgroup *1* at ZT14	***O***—0.5 mL peanut butter flavouring (Uncle Roy's Comestible Concoctions, Ayr, Scotland) in a brown glass vial
		Subgroup *2* at ZT02	***P***—0.5 mL cheese flavouring (Uncle Roy's Comestible Concoctions, Ayr, Scotland) in a brown glass vial

Note—*Subgroup *1*: $n = 6$; subgroup *2*: $n = 5$. †The identity of novel and familiar stimuli and their spatial positions at test were counterbalanced within each subgroup, to take into account any potential bias toward a certain stimulus or part of the arena, e.g., for half of each subgroup object ***A*** was assigned as the familiar stimulus and object ***B*** as the novel stimulus at test; this arrangement was reversed for the remaining half of each subgroup, so that object ***B*** was familiar and object ***A*** was novel at test; in addition, the novel stimulus was located at the top left corner of the arena at test for half of each subgroup, whereas the novel stimulus was located at the bottom right corner in the remaining cases. The same counterbalancing of object/odour stimuli was applied to subgroups *1* and *2* under both lighting conditions. ‡Each brown glass vial had a base diameter 1.5 cm × height 2.2 cm, with a circular opening 0.7 cm diameter.

Table S4. Mean Recognition Scores (± Standard Error of the Mean) Across the Three Minutes of the Test Phase

ZT	LIGHTING	MINUTE OF TEST 0–60 s	60–120 s	60–180 s	POOLED 0–180 s
ZT02	LD	.780 ± .048	.562 ± .102	.486 ± .084	.609 ± .050
	DLE	.672 ± .057	.419 ± .076	.662 ± .068	.582 ± .033
ZT14	LD	.580 ± .071	.678 ± .051	.489 ± .062	.585 ± .036
	DLE	.852 ± .052	.540 ± .067	.541 ± .086	.644 ± .039
Main effect of Lighting:		F=2.263, p=.163	F=4.235, p=.067	F=5.312, p=.044*	F=.244, p=.632
Lighting × Time of Day:		F=9.879, p=.01*	F=.002, p=.965	F=1.055, p=.328	F=4.026, p=.073

Note—Recognition scores were expressed as the proportion of time spent with the novel stimulus [*novel*/(*novel* + *familiar*)]. *Significant effects from 2-way within-subjects ANOVAs (ps < .05).

Table S5. Gene Stability Indices for Housekeeping Genes *Arbp*, *Tbp*, and *Gapdh*

TISSUE	LIGHTING	HOUSEKEEPING GENE		
		Arbp	*Tbp*	*Gapdh*
Heart	LD	.80	.74	.73
	DLE	4.36	2.87	3.22
Liver	LD	.85	1.20	.90
	DLE	4.03	3.62	3.99
Adrenal gland	LD	.62	.56	.57
	DLE	3.61	2.72	3.31
Dorsal hippocampus	LD	.57	.64	.46
	DLE	3.76	2.84	3.18
POOLED		2.32	1.90	2.05

Table S6. *P*-values from One-way ANOVAs Examining Effects of Time of Day on Housekeeping Gene Expression

TISSUE	LIGHTING	HOUSEKEEPING GENE			$NF_2^{(Tbp,Gapdh)}$
		Arbp	*Tbp*	*Gapdh*	
Heart	LD	.608	.401	.513	.558
	DLE	.994	.493	.001*	.121
Liver	LD	.481	.033*	.229	.132
	DLE	.571	.353	.460	.570
Adrenal gland	LD	.330	.058	.163	.059
	DLE	.937	.654	.007*	.417
Dorsal hippocampus	LD	.214	.006*	.017*	.005*
	DLE	.687	.107	.111	.904

Note—*Significant main effects from 1-way between-subjects ANOVAs (*p*s < .05). Data are not corrected for multiple comparisons. NF_2 = normalisation factor calculated from *Tbp* and *Gapdh*.

Table S7. Acrophase of Clock Gene Expression Under LD: A Comparison Between Published Studies and the Current Study

GENE	TISSUE	PUBLISHED	THIS STUDY	Δ (h)
Per2	Heart	ZT12 [*a*]	ZT14	+2
	Liver	ZT12 [*a*]	ZT14	+2
	Adrenal gland	ZT14 [*b*]	ZT14	0
	Dorsal hippocampus	ZT16 [*c*]	ZT14	−2
Bmal1	Heart	ZT00 [*a*]	ZT02	+2
	Liver	ZT00 [*a*]	ZT02	+2
	Adrenal gland	ZT22 [*b*]	ZT20	−2
	Dorsal hippocampus	ZT03 [*c*]	ZT02	−1
Rev-erbα	Heart	ZT08 [*d*]	ZT02	−6
	Liver	ZT08 [*d*]	ZT08	0
	Adrenal gland	ZT06 [*b*]	ZT02	−4
	Dorsal hippocampus	ZT06 [*e*]	ZT08	+2
Cry1	Heart	ZT12 [*a*]	ZT14	+2
	Liver	ZT12 [*a*]	ZT14	+2
	Adrenal gland	ZT18 [*b*]	ZT14	−4
	Dorsal hippocampus	ZT00 [*f*]	ZT02	+2
Dbp	Heart	ZT08 [*d*]	ZT08	0
	Liver	ZT08 [*d*]	ZT08	0
	Adrenal gland	ZT09 [*g*]	ZT08	−1
	Dorsal hippocampus	ZT10 [*c*]	ZT08	−2

Note—[*a*] Peirson, S. N. *et al*. (2006). *Biochemical and biophysical research communications*, *351*(4), 800–807; [*b*] Oster, H. *et al*. (2006). *Cell metabolism*, *4*(2), 163–173; [*c*] Harbour, V. L. *et al*. (2014). *PloS one*, *9*(7), e103309; [*d*] Bonaconsa, M. *et al*. (2014). *Experimental gerontology*, *55*, 70–79; [*e*] Valnegri, P. *et al*. (2011). *Nature neuroscience*, *14*(10), 1293–1301; [*f*] Tischkau, S. A. *et al*. (2007). *Experimental neurology*, *208*(2), 314–322; [*g*] Watanabe, T. *et al*. (2006). *Neuroscience research*, *54*(4), 295–301. Difference (Δ) = acrophase in the current study − acrophase in published studies. In the current study, tissue samples were collected every 6 h starting from ZT02, so exact agreement in acrophase only occurs where published studies used identical sampling ZTs.

SUPPLEMENTARY FIGURES S1–S11

Fig. S1. Wavelet analyses of PIR locomotor activity under DLE

Fig. S2. Effects of DLE on skin temperature measured by infrared thermography

Fig. S3. Effects of DLE *versus* 16:8 LD on locomotor activity and immobility-defined sleep

Fig. S4. Phase shifts in molecular clock rhythms under DLE

Fig. S5. Liver metabolic gene expression and body weight under DLE

Fig. S6. Effects of DLE on short-term recognition memory performance and stimulus exploration

Fig. S7. Dissociation of SCN subregional cFos signals under DLE

Fig. S8. cFos signals in superficial and deep layers of the prefrontal cortex under DLE

Fig. S9. cFos signals in the preoptic hypothalamus under DLE

Fig. S10. cFos signals in the lateral and dorsomedial hypothalamus under DLE

Fig. S11. Dopaminergic signals in the hypothalamus and midbrain under DLE

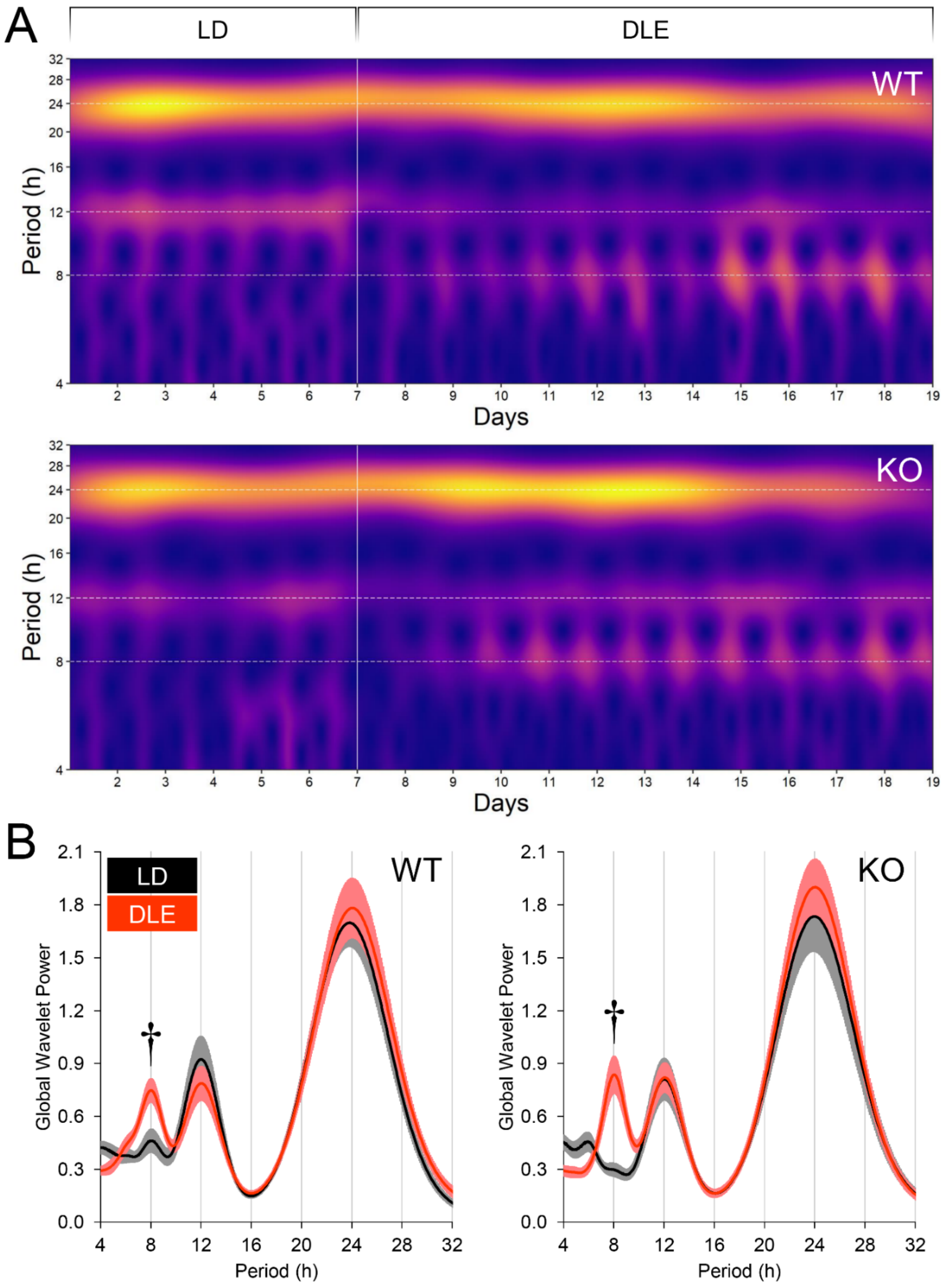

Fig. S1. Wavelet analyses of PIR locomotor activity under DLE. Panel **A** shows the group-level, time (day)–frequency (period length) spectra under LD and DLE; the day of transition from LD to DLE is marked by the white vertical line. Brighter (*yellow*) colours indicate higher local wavelet power, whereas darker (*purple*) colours indicate lower local wavelet power. Period lengths on the y-axis are plotted on a 2^y scale. Panel **B** shows the global wavelet power at each period length, pooling data across the time domain ($N = 12$ WT and 12 $Opn4^{-/-}$ mice). Under DLE, the power of ~24 h oscillations persisted in both genotypes. However, there was an increase in oscillatory power at ~8 h (†main effects of Lighting $ps < .01$). This is likely to be an artefact of the compressed (8-h) night under DLE, rather than reflecting an increase in ultradian rhythms *per se*.

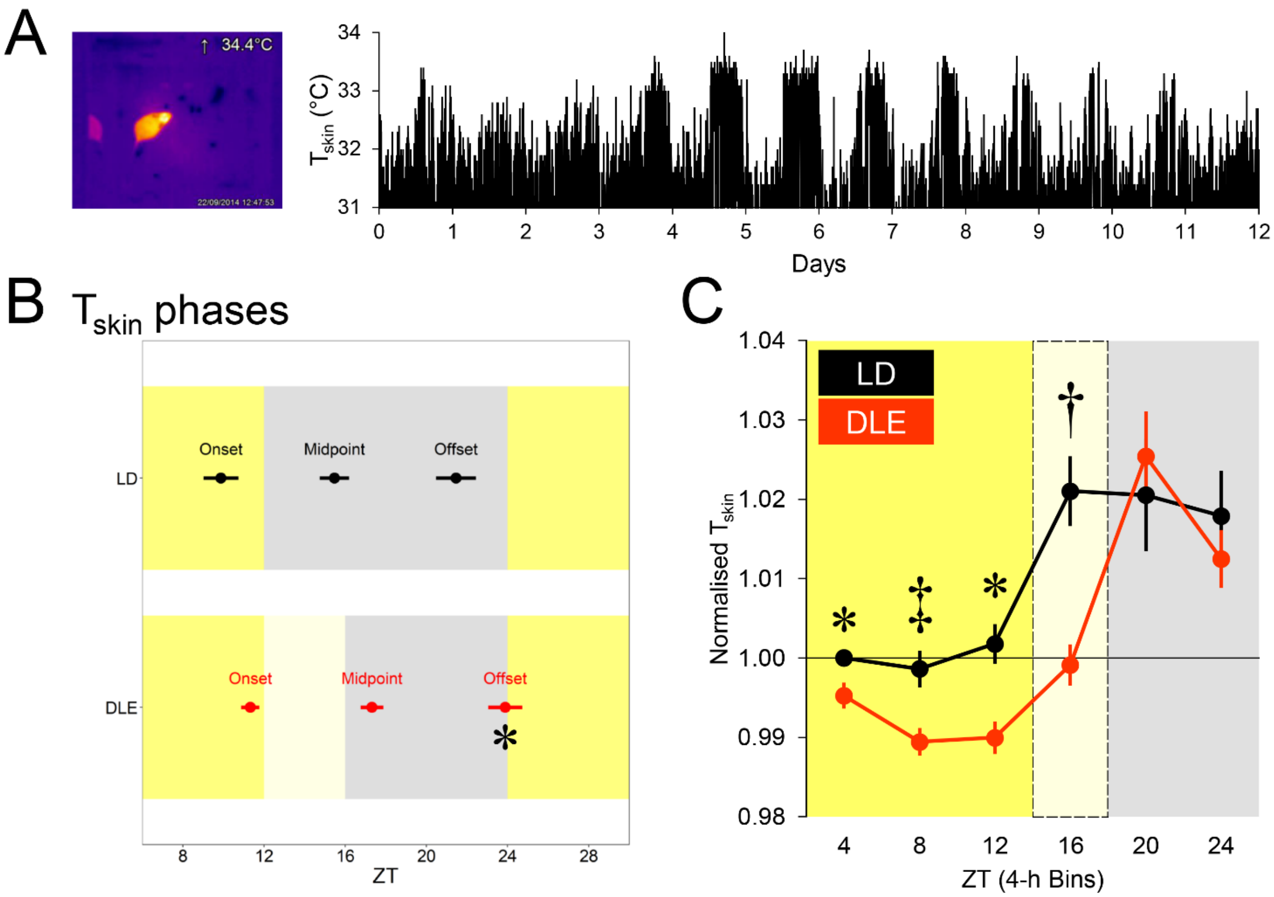

Fig. S2. Effects of DLE on skin temperature (T_{skin}) measured by infrared thermography (*N* = 5 WT mice). Panel **A**, *left*, shows a screenshot of the thermal imaging recording. Brighter (*yellow*) colours indicate higher temperature, whereas darker (*purple*) colours indicate lower temperature. Panel **A**, *right*, shows 6 days of T_{skin} recording under LD followed by 6 days under DLE from one mouse. Panel **B** shows T_{skin} onsets, midpoints, and offsets. Zeitgeber times on the x-axis are plotted from ZT6 to ZT30. T_{skin} offsets, but not onsets and midpoints, were significantly delayed under DLE (*main effect of Lighting $p = .046$). Panel **C** shows normalised T_{skin} data in 4-h bins; data from each mouse are normalised to the value in the first 4-h bin under LD. Under DLE, there was a 0.5–1% reduction in T_{skin} (~0.15–0.35°C) in the light phase and a 2% drop (~0.65°C) during the DLE period (main effect of Lighting $p = .011$; Lighting × Time of Day interaction $p = .016$; simple effects of Lighting: *$ps < .05$; †$p < .01$; ‡$p < .005$).

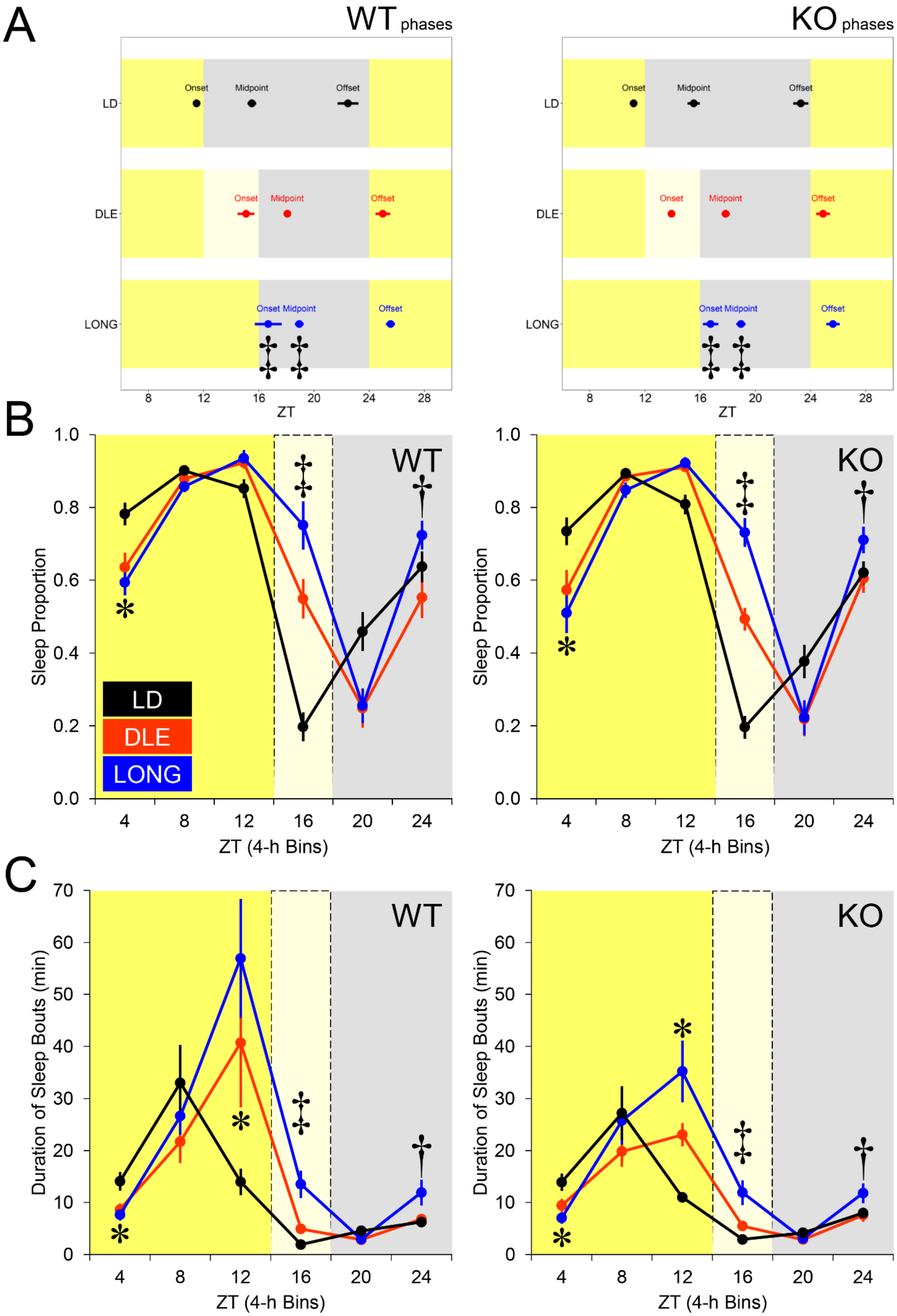

Fig. S3. Effects of DLE (*red*) *versus* 16:8 LD (long-day photoperiods, *blue*) on PIR locomotor activity and immobility-defined sleep (N = 12 WT and 12 *Opn4*$^{-/-}$ mice). Effects of evening light are light-intensity dependent. Panel **A** shows activity onsets, midpoints, and offsets. Zeitgeber times on x-axes are plotted from ZT6 to ZT30. Activity onsets and midpoints, but not offsets, were further delayed under 16:8 LD relative to DLE [‡main effects of Lighting (16:8 LD *vs*. DLE) $ps < .001$]. Panels **B** and **C** show that effects of evening light on sleep proportion and duration of sleep bouts were enhanced under 16:8 LD [simple effects of Lighting (16:8 LD *vs*. DLE): *$ps < .05$; †$ps < .005$; ‡$ps < .001$].

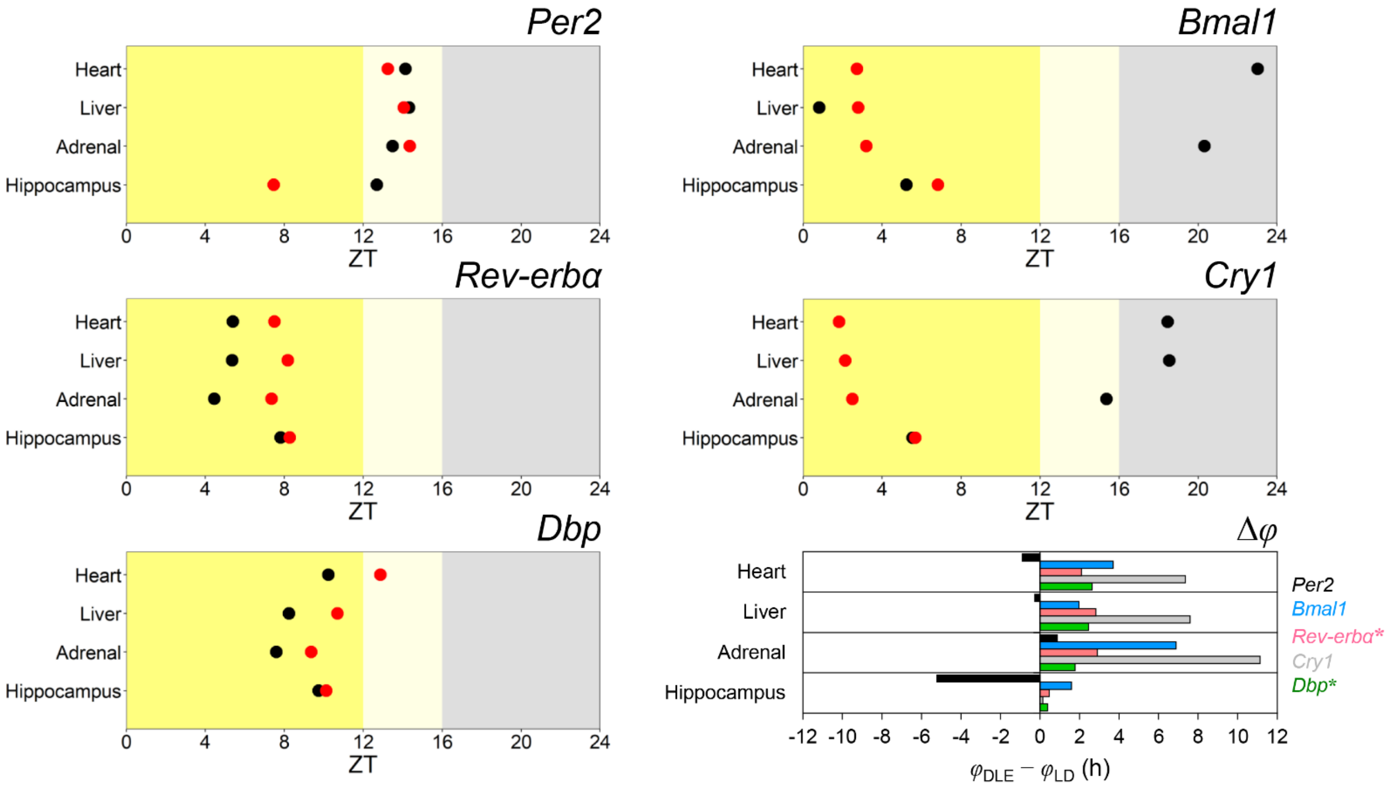

Fig. S4. Phase shifts in molecular clock rhythms under DLE. Centres of gravity (CoG) were obtained from the CircWave software [2,3,4], providing an estimate of the acrophase (φ) of the molecular rhythm (CoG under LD, *black* circles; CoG under DLE, *red* circles). Phase shifts in clock gene expression under DLE, $\Delta\varphi = \varphi_{DLE} - \varphi_{LD}$, are summarised in the *bottom right* panel [*one-sample *t* tests comparing means *vs*. the value of 0, *p*s < .05 (2-tailed)].

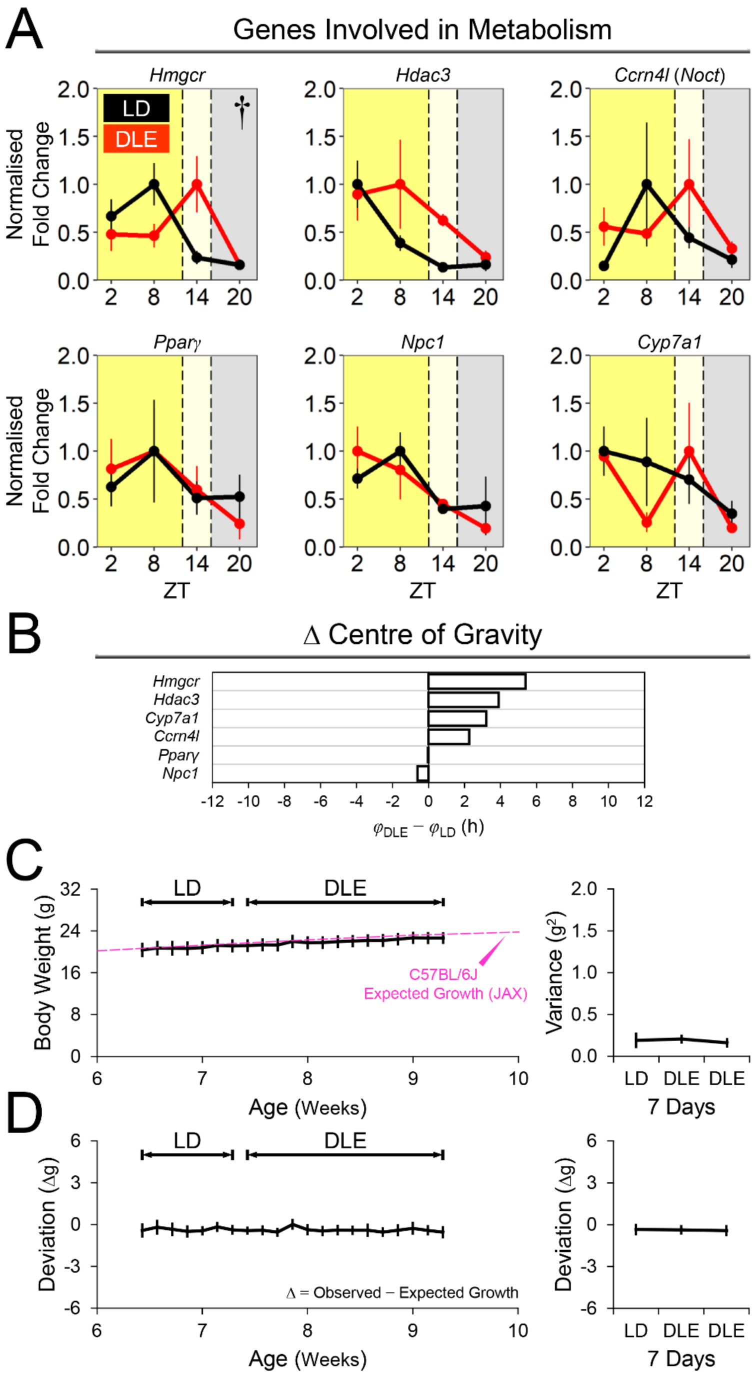

Fig. S5. Liver metabolic gene expression and body weight under DLE. Panel **A** shows that the peak of hepatic *Hmgcr* expression was delayed under DLE (†Lighting × Time of Day interaction $p = .008$); there was no significant Lighting × Time of Day interaction involving *Hdac3*, *Ccrn4l* (*Noct*), *Pparγ*, *Npc1*, or *Cyp7a1* ($N = 32$ WT mice in total; 4 mice per condition). Data are normalised to peak expression values in each lighting condition. Panel **B** shows shifts in the centre of gravity ($\Delta\varphi = \varphi_{DLE} - \varphi_{LD}$) of hepatic metabolic rhythms under DLE. The mean $\Delta\varphi$ of all 6 genes was 2.35 ± .95 h [one-sample *t* test comparing mean *vs*. the value of 0, $p = 0.055$ (2-tailed)]. Panel **C**, *left*, shows daily body weights under 7 days of LD and 2 weeks of DLE ($N = 8$ WT mice; 4♀ and 4♂ mice); mice were fed with Teklad 2916 diet containing 16% protein and 4% fat. Observed body weights are plotted against the C57BL/6J mouse's expected growth curve (*magenta dashed* line), which shows average body weights of up to 360♀ and 360♂ mice fed with LabDiet 5K52 containing 19% protein and 6% fat [the Jackson Laboratory, JAX stock 000664; 5]. Panel **C**, *right*, shows inter-day variability in body weights during each week; there was no effect of DLE ($p = .709$). Panel **D** shows daily (*left*) and weekly deviation (*right*) of observed body weights from the C57BL/6J mouse's expected growth curve. There was no effect of DLE ($p = .892$), and mean deviation values (pooled across 7 days) were not different from the value of 0 during the entire 3-week period [one-sample *t* tests (2-tailed): LD, $p = .317$; 1st week of DLE, $p = .150$; 2nd week of DLE, $p = .238$].

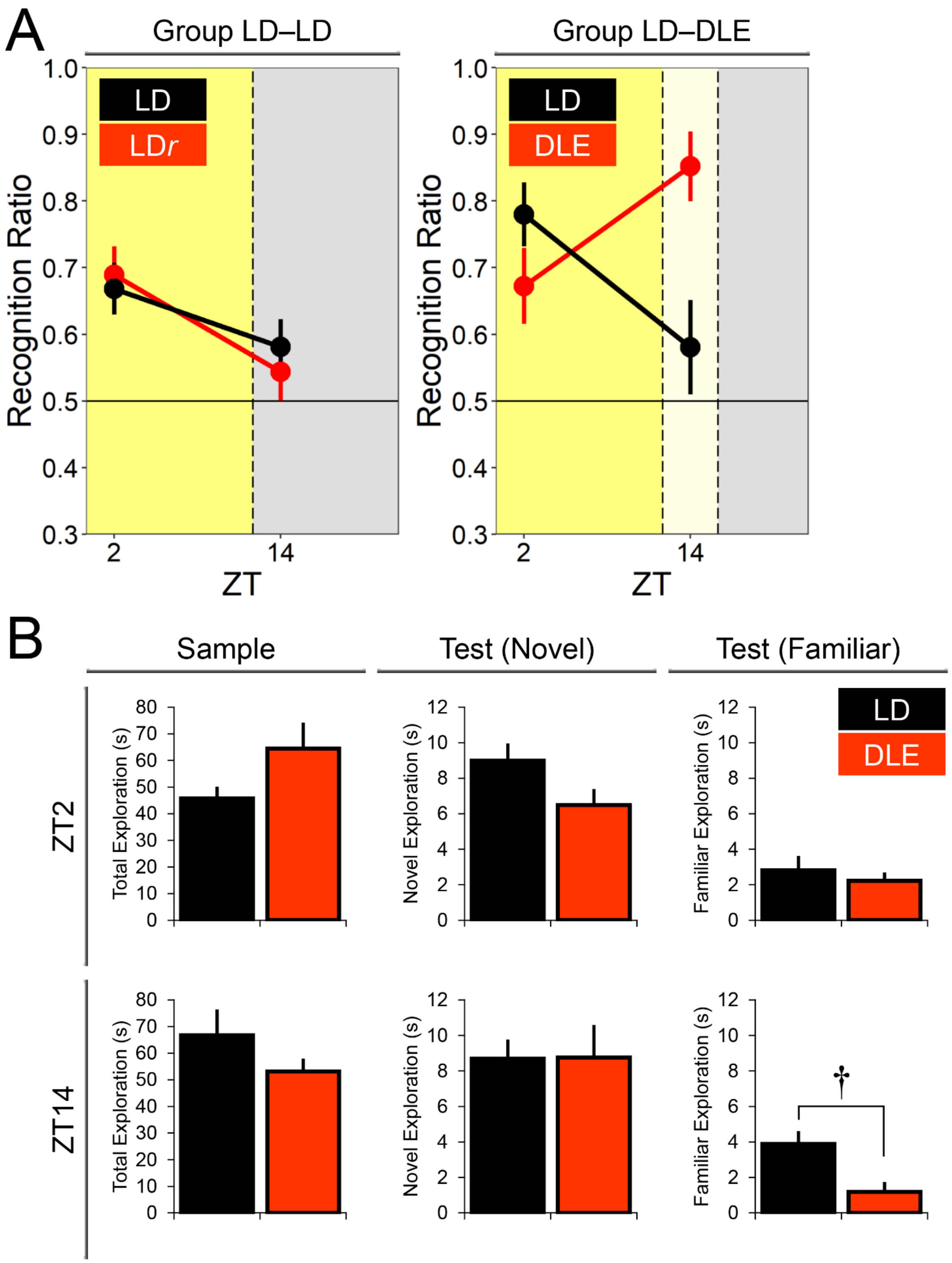

Fig. S6. Effects of DLE on short-term recognition memory performance and stimulus exploration. Panel **A**, *left*, shows recognition ratios from Group LD–LD ($N = 16$ WT mice), which received recognition trials at ZT2 and ZT14 under LD and their recognition performance was reassessed 2 weeks later (LD*r*) at the same Zeitgeber times. In these mice, there was a main effect of Time of Day ($p < 0.05$) but no effect of Repeated Testing (main effect of Repeated Testing $p = .849$; Time of Day × Repeated Testing interaction $p = .449$). Thus, in contrast to the reversed performance in Group LD–DLE ($N = 11$ WT mice; *right*), repeated testing in Group LD–LD did not alter the behavioural rhythm in recognition memory performance. Panel **B** shows stimulus exploration times during sample and test phases in Group LD–DLE (pooled across object and odour trials). Under DLE at ZT14, the amount of time spent exploring the familiar stimulus at test was reduced (†main effect of Lighting $p = .007$).

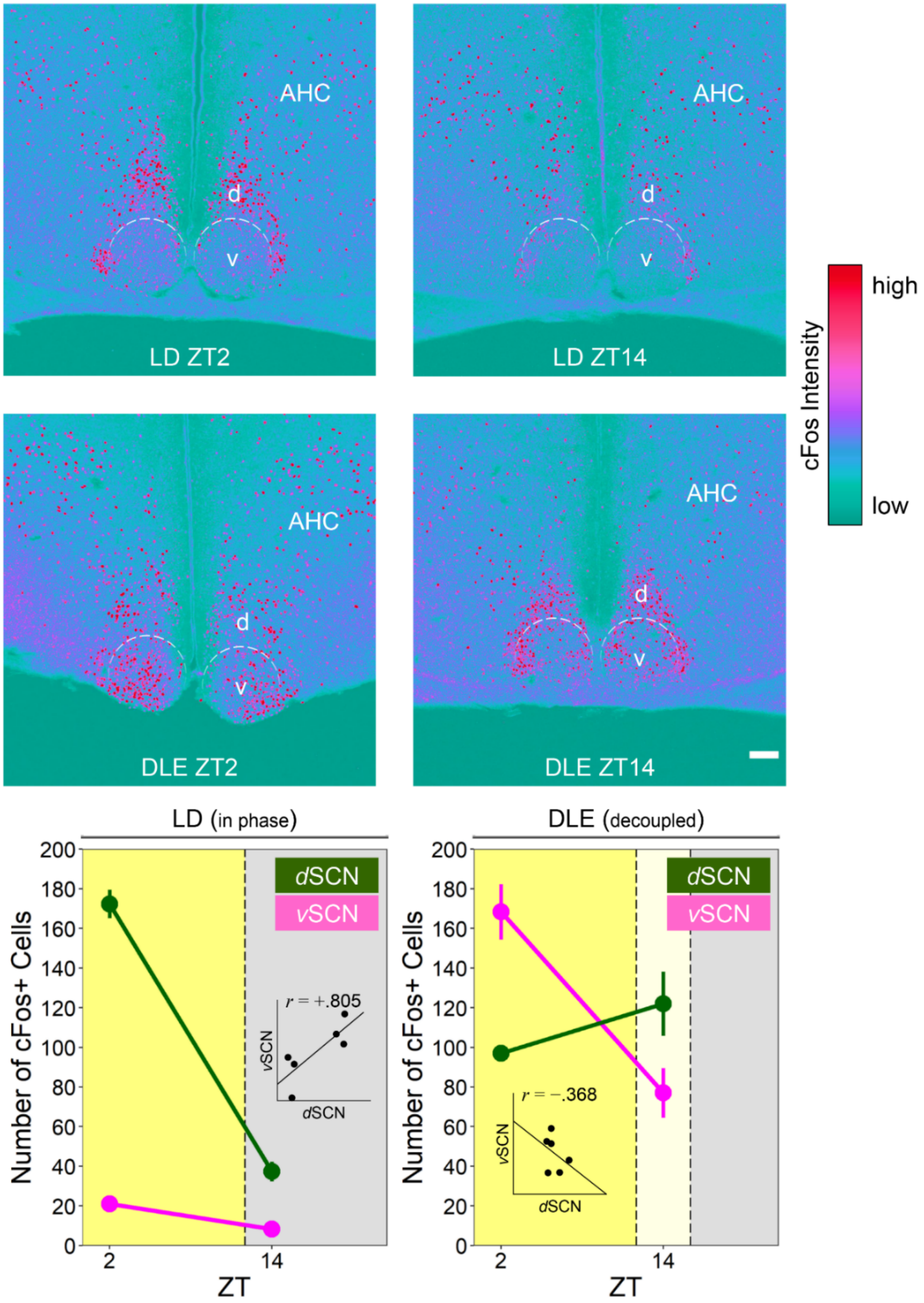

Fig. S7. Dissociation of SCN subregional cFos signals under DLE (N = 3 WT mice per condition). Representative images show immunofluorescence cFos+ cells in the dorsal SCN (*d*SCN; shell) and ventral SCN (*v*SCN; core). Warmer colours (*red*) indicate higher cFos intensity, whereas colder colours (*cyan*) indicate lower cFos intensity. Coronal sections are Bregma −0.82 mm in Franklin and Paxinos' atlas [6], corresponding to plate 100048576_241 in the Allen Mouse Brain Atlas [7]. AHC indicates the anterior hypothalamic area, central part. The white scale bar represents 100 µm. Under LD (*bottom left*), there were more cFos+ cells in the *d*SCN than in the *v*SCN, but in both subregions cFos+ cell counts were higher at ZT2 than at ZT14, resembling the pattern reported in a previous study [8]. By contrast, under DLE (*bottom right*) cFos signals were reversed in the *d*SCN, whereas in the *v*SCN cFos levels were upregulated without any change in phasing (Lighting × Time of Day × Subregion interaction $p < .001$). *Insets* show that *d*SCN and *v*SCN cFos+ cell counts were positively correlated under LD (p = .053) but not under DLE (p = .473).

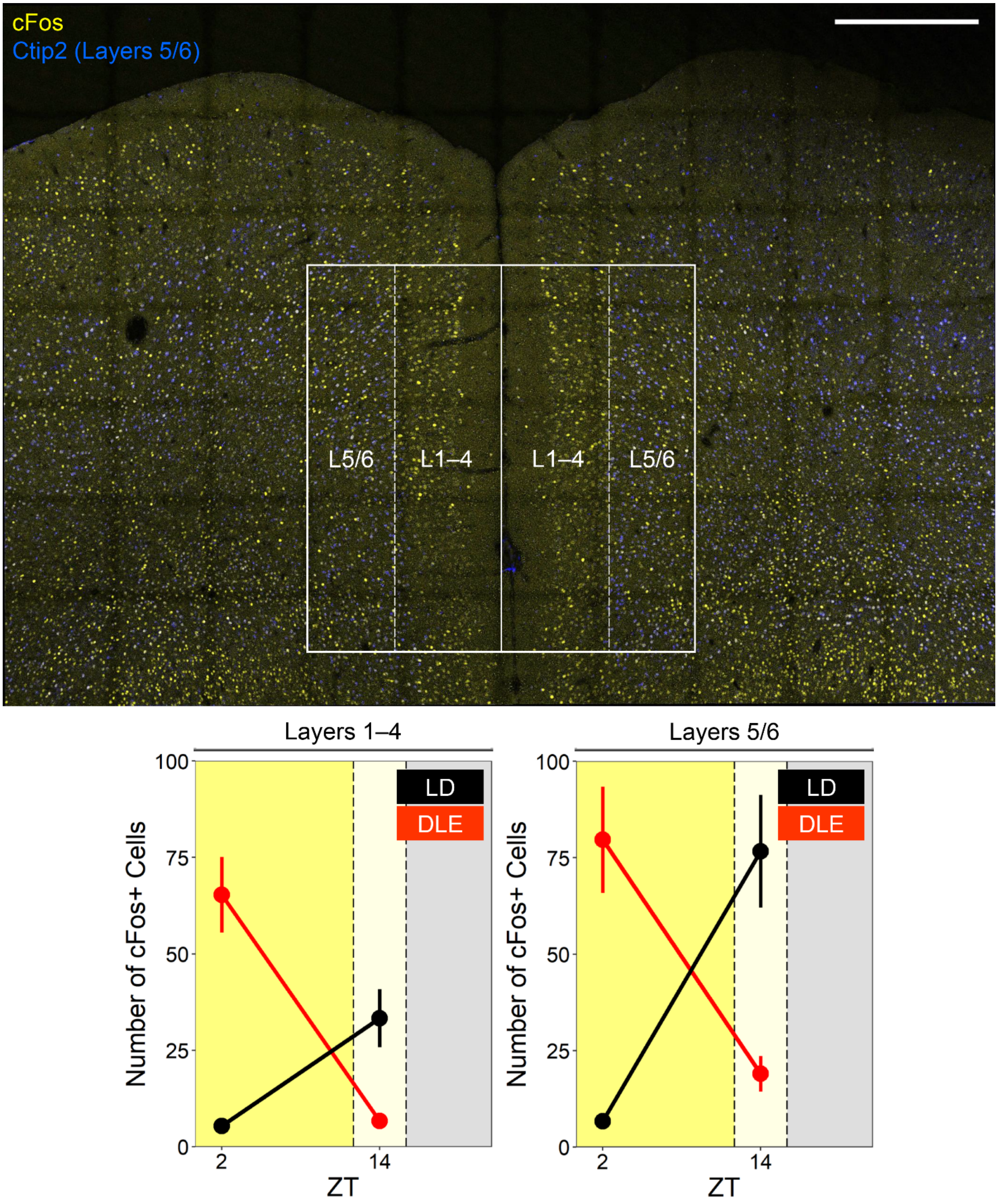

Fig. S8. Reversal of cFos signals in superficial (layers 1–4) and deep layers (layers 5/6) of the prelimbic/infralimbic cortex (*N* = 3 WT mice per condition). Ctip2 (*blue*) marks cortical layers 5/6. Coronal sections are near Bregma +1.98 mm in Franklin and Paxinos' atlas [6], corresponding to plate 100048576_117 in the Allen Mouse Brain Atlas [7]. The white scale bar represents 500 μm. Reversal of cFos signals was found in both superficial and deep cortical layers (Lighting × Time of Day interactions *p*s $< .001$; Lighting × Time of Day × Cortical Layer interaction $p = .117$).

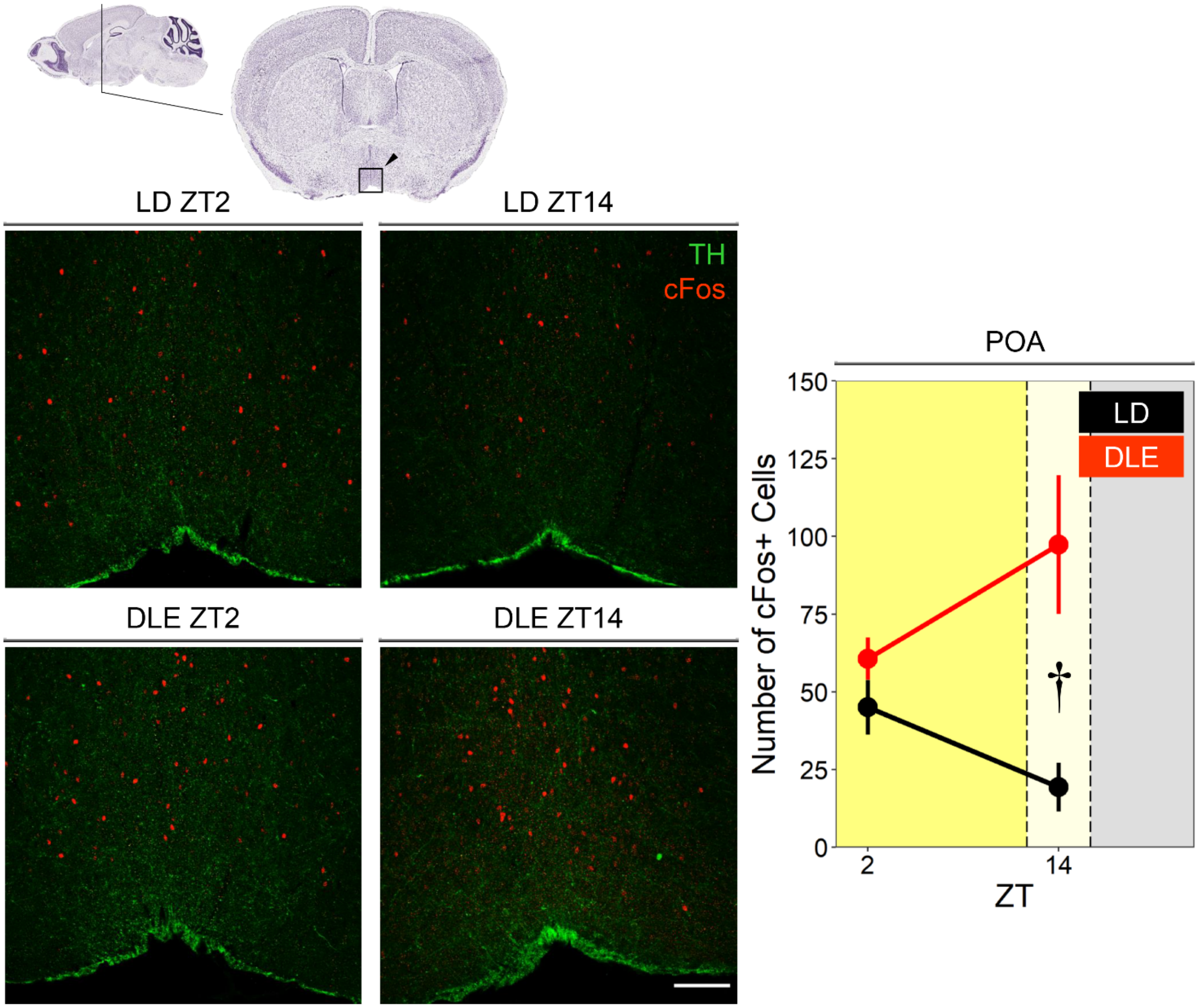

Fig. S9. cFos signals in the preoptic hypothalamus under DLE (*N* = 3 WT mice per condition). Representative images from the four conditions show immunofluorescence cFos-positive (cFos+; *red*) cells in the preoptic regions (POA)—which contain putative thermoregulatory neurons [9,10,11] as well as putative sleep-regulatory neurons [12,13]—including the median preoptic nucleus (MnPO), ventromedial preoptic nucleus (VMPO), ventrolateral preoptic nucleus (VLPO), and medial preoptic area (MPA). Coronal sections are near Bregma +0.14 mm in Franklin and Paxinos' atlas [6], corresponding to plate 100048576_209 retrieved from the Allen Mouse Brain Atlas [7]. The white scale bar represents 100 μm. The number of cFos+ cells in the POA was increased at ZT14 (main effect of Lighting $p = .007$; Lighting × Time of Day interaction $p = .044$; †simple effect of Lighting $p = .003$), corresponding to the drop in body temperature (**Fig. S2C**) and increased sleep during the DLE period (**Fig. 2B**).

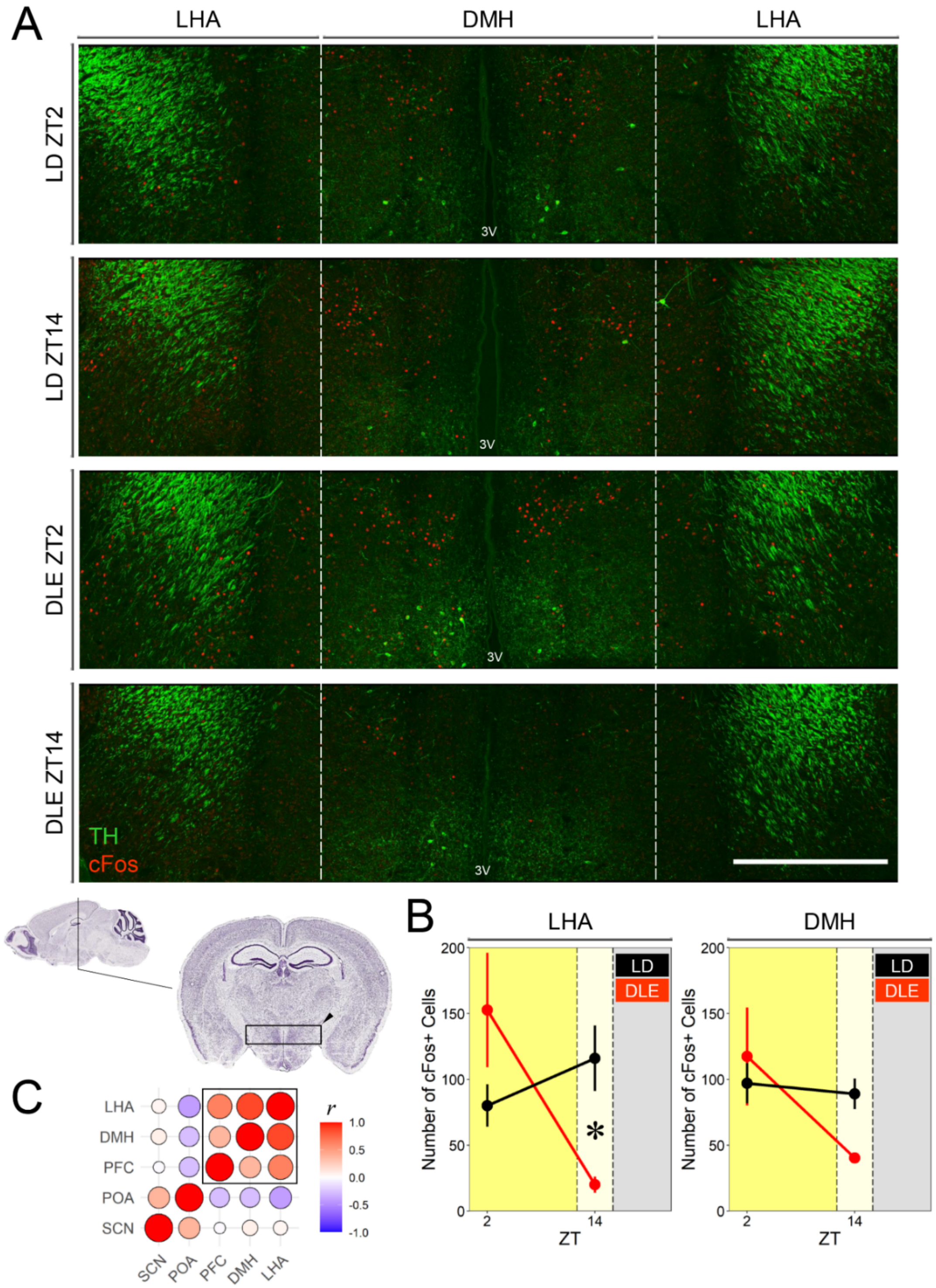

Fig. S10. cFos signals in the lateral hypothalamic area (LHA) and dorsomedial hypothalamic nucleus (DMH) under DLE (*N* = 3 WT mice per condition). Panel **A** shows representative images of immunofluorescence cFos-positive (cFos+; *red*) cells in the LHA and DMH, which contain putative wakefulness-promoting neurons [14,15,16]. The LHA is densely innervated by tyrosine-hydroxylase-positive (TH+; *green*) fibres. Coronal sections are near Bregma −1.94 mm in Franklin and Paxinos' atlas [6], corresponding to plate 100048576_281 retrieved from the Allen Mouse Brain Atlas [7]. The white scale bar represents 500 µm. 3V indicates the 3rd ventricle. Panel **B** shows that the number of cFos+ cells in the LHA was reduced during the DLE period (Lighting × Time of Day interaction $p = .013$; *simple effect of Lighting at ZT14 $p = .033$), in contrast to the POA (**Fig. S9**). Panel **C** shows the correlation matrix of cFos+ cell counts between different brain regions. cFos+ cell counts in the LHA and DMH were positively related to each other and to PFC (*red* circles clustered inside the square), but these were negatively related to the POA (*blue* circles).

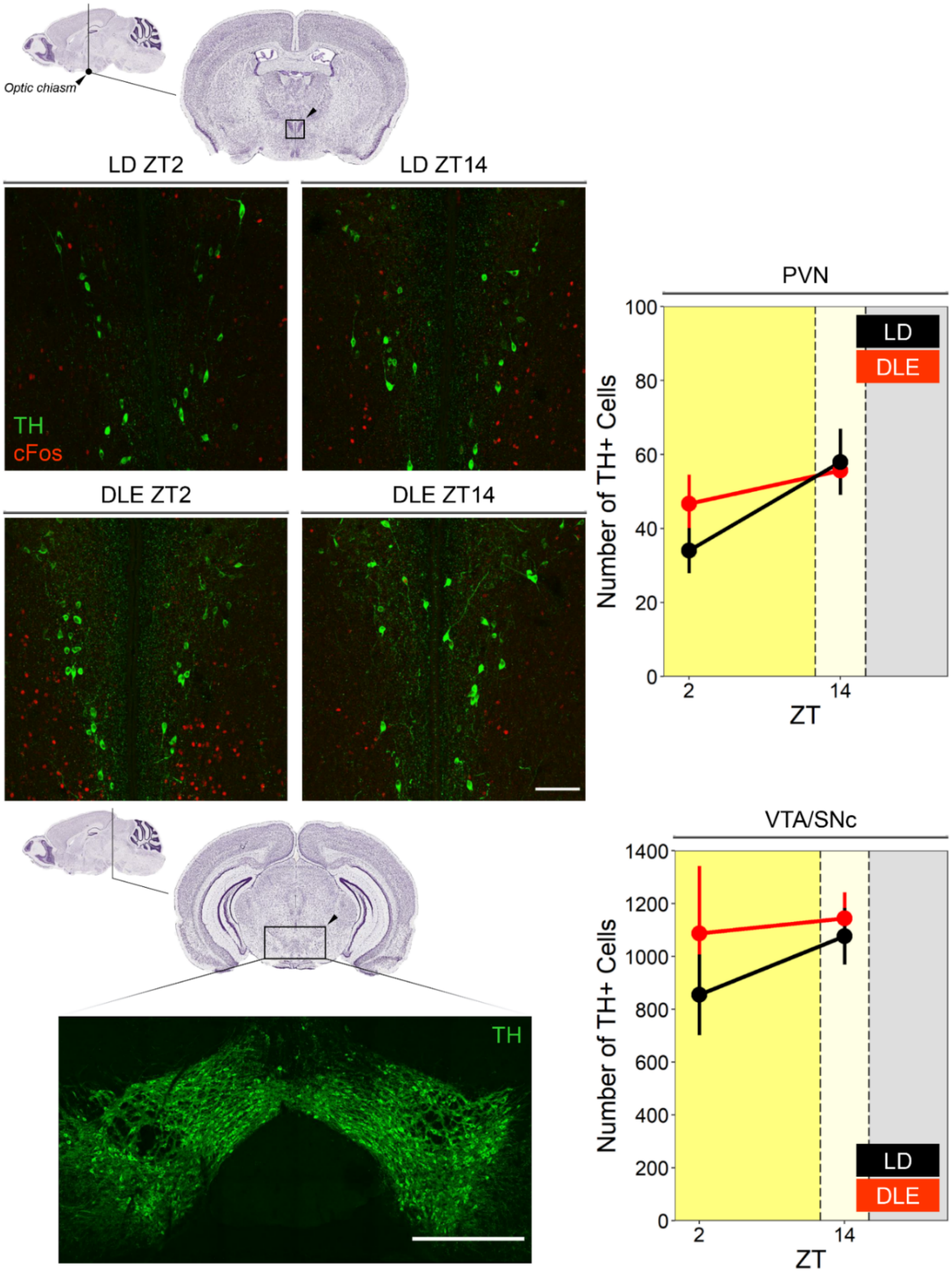

Fig. S11. Dopaminergic signals in the hypothalamus and midbrain under DLE (N = 3 WT mice per condition). *Upper* panels show representative images of immunofluorescence tyrosine-hydroxylase expressing (TH+; *green*) cells in the paraventricular nuclei of the hypothalamus (PVN). Coronal sections are near Bregma −0.82 mm in Franklin and Paxinos' atlas [6], corresponding to plate 100048576_241 retrieved from the Allen Mouse Brain Atlas [7]. The white scale bar represents 100 μm. Unlike long-day photoperiods, DLE did not alter TH+ cell counts in the PVN (main effect of Lighting p = .495; Lighting × Time of Day interaction p = .330). When pooled across Lighting conditions there were more TH+ cells at ZT14 than at ZT2 (main effect of Time of Day p = .052), consistent with the nocturnal pattern of TH expression observed in the pineal gland [17], striatum [18,19], and midbrain [20]. *Lower* panels show that TH+ cell counts in the midbrain, including the ventral tegmental area (VTA) and substantia nigra pars compacta (SNc), were unaffected under DLE (main effect of Lighting p = .391; Lighting × Time of Day interaction p = .635). The coronal section is near Bregma −3.16 mm in Franklin and Paxinos' atlas [6], corresponding to plate 100048576_342 retrieved from the Allen Mouse Brain Atlas [7]. The white scale bar represents 500 μm.

SUPPLEMENTARY REFERENCES

[1] Vandesompele, J., De Preter, K., Pattyn, F., Poppe, B., Van Roy, N., De Paepe, A., & Speleman, F. (2002). Accurate normalization of real-time quantitative RT-PCR data by geometric averaging of multiple internal control genes. *Genome biology*, *3*(7), RESEARCH0034.

[2] Oster, H., Damerow, S., Hut, R. A., & Eichele, G. (2006). Transcriptional profiling in the adrenal gland reveals circadian regulation of hormone biosynthesis genes and nucleosome assembly genes. *Journal of Biological Rhythms*, *21*(5), 350–361.

[3] Hut, R. A. (2007). *CircWave version 1.4*. https://www.euclock.org/results/item/circ-wave.html

[4] van der Veen, D. R., Mulder, E. G., Oster, H., Gerkema, M. P., & Hut, R. A. (2008). SCN-AVP release of *mPer1/mPer2* double-mutant mice *in vitro*. *Journal of Circadian Rhythms*, *6*, 5.

[5] The Jackson Laboratory (2021). *Body Weight Information for C57BL/6J (00064)*. https://www.jax.org/jax-mice-and-services/strain-data-sheet-pages/body-weight-chart-000664

[6] Franklin, K. B. J. & Paxinos, G. (2007). *The Mouse Brain in Stereotaxic Coordinates*, 3rd edition. Academic Press.

[7] Lein, E. S., Hawrylycz, M. J., Ao, N., Ayres, M., Bensinger, A., Bernard, A., Boe, A. F., Boguski, M. S., Brockway, K. S., Byrnes, E. J., Chen, L., Chen, L., Chen, T. M., Chin, M. C., Chong, J., Crook, B. E., Czaplinska, A., Dang, C. N., Datta, S., Dee, N. R., … & Jones, A. R. (2007). Genome-wide atlas of gene expression in the adult mouse brain. *Nature*, *445*(7124), 168–176.

[8] Duy, P. Q., Komal, R., Richardson, M., Hahm, K. S., Fernandez, D. C., & Hattar, S. (2020). Light has diverse spatiotemporal molecular changes in the mouse suprachiasmatic nucleus. *Journal of Biological Rhythms*, *35*(6), 576–587.

[9] Zhao, Z. D., Yang, W. Z., Gao, C., Fu, X., Zhang, W., Zhou, Q., Chen, W., Ni, X., Lin, J. K., Yang, J., Xu, X. H., & Shen, W. L. (2017). A hypothalamic circuit that controls body temperature. *Proceedings of the National Academy of Sciences of the United States of America*, *114*(8), 2042–2047.

[10] Harding, E. C., Yu, X., Miao, A., Andrews, N., Ma, Y., Ye, Z., Lignos, L., Miracca, G., Ba, W., Yustos, R., Vyssotski, A. L., Wisden, W., & Franks, N. P. (2018). A neuronal hub binding sleep initiation and body cooling in response to a warm external stimulus. *Current Biology*, *28*(14), 2263–2273.e4.

[11] Wang, T. A., Teo, C. F., Åkerblom, M., Chen, C., Tynan-La Fontaine, M., Greiner, V. J., Diaz, A., McManus, M. T., Jan, Y. N., & Jan, L. Y. (2019). Thermoregulation via temperature-dependent PGD_2 production in mouse preoptic area. *Neuron*, *103*(2), 309–322.e7.

[12] Chung, S., Weber, F., Zhong, P., Tan, C. L., Nguyen, T. N., Beier, K. T., Hörmann, N., Chang, W. C., Zhang, Z., Do, J. P., Yao, S., Krashes, M. J., Tasic, B., Cetin, A., Zeng, H., Knight, Z. A., Luo, L., & Dan, Y. (2017). Identification of preoptic sleep neurons using retrograde labelling and gene profiling. *Nature*, *545*(7655), 477–481.

[13] Kroeger, D., Absi, G., Gagliardi, C., Bandaru, S. S., Madara, J. C., Ferrari, L. L., Arrigoni, E., Münzberg, H., Scammell, T. E., Saper, C. B., & Vetrivelan, R. (2018). Galanin neurons in the ventrolateral preoptic area promote sleep and heat loss in mice. *Nature Communications*, *9*(1), 4129.

[14] Rolls, A., Colas, D., Adamantidis, A., Carter, M., Lanre-Amos, T., Heller, H. C., & de Lecea, L. (2011). Optogenetic disruption of sleep continuity impairs memory consolidation. *Proceedings of the National Academy of Sciences of the United States of America*, *108*(32), 13305–13310.

[15] Piñol, R. A., Zahler, S. H., Li, C., Saha, A., Tan, B. K., Škop, V., Gavrilova, O., Xiao, C., Krashes, M. J., & Reitman, M. L. (2018). Brs3 neurons in the mouse dorsomedial hypothalamus regulate body temperature, energy expenditure, and heart rate, but not food intake. *Nature Neuroscience*, *21*(11), 1530–1540.

[16] Naganuma, F., Kroeger, D., Bandaru, S. S., Absi, G., Madara, J. C., & Vetrivelan, R. (2019). Lateral hypothalamic neurotensin neurons promote arousal and hyperthermia. *PLoS Biology*, *17*(3), e3000172.

[17] McGeer, E. G. & McGeer, P. L. (1966). Circadian rhythm in pineal tyrosine hydroxylase. *Science*, *153*(3731), 73–74.

[18] Sleipness, E. P., Sorg, B. A., & Jansen, H. T. (2007). Diurnal differences in dopamine transporter and tyrosine hydroxylase levels in rat brain: Dependence on the suprachiasmatic nucleus. *Brain Research*, *1129*(1), 34–42.

[19] Webb, I. C., Baltazar, R. M., Wang, X., Pitchers, K. K., Coolen, L. M., & Lehman, M. N. (2009). Diurnal variations in natural and drug reward, mesolimbic tyrosine hydroxylase, and clock gene expression in the male rat. *Journal of Biological Rhythms*, *24*(6), 465–476.

[20] Logan, R. W., Parekh, P. K., Kaplan, G. N., Becker-Krail, D. D., Williams, W. P., 3rd, Yamaguchi, S., Yoshino, J., Shelton, M. A., Zhu, X., Zhang, H., Waplinger, S., Fitzgerald, E., Oliver-Smith, J., Sundarvelu, P., Enwright, J. F., 3rd, Huang, Y. H., & McClung, C. A. (2019). NAD+ cellular redox and SIRT1 regulate the diurnal rhythms of tyrosine hydroxylase and conditioned cocaine reward. *Molecular Psychiatry*, *24*(11), 1668–1684.
